# Determinants of Response to Immune Checkpoint Blockade in Pleural Mesothelioma: Molecular, Immunological, and Clinical Perspectives

**DOI:** 10.3390/cancers17244020

**Published:** 2025-12-17

**Authors:** Martina Delsignore, Gaia Cassinari, Simona Revello, Luigi Cerbone, Federica Grosso, Marcello Arsura, Chiara Porta

**Affiliations:** 1 Department of Pharmaceutical Sciences, Università del Piemonte Orientale “Amedeo Avogadro”, 28100 Novara, Italy; martina.delsignore@uniupo.it (M.D.); gaia.cassinari@uniupo.it (G.C.); 2 Center for Translational Research on Autoimmune & Allergic Diseases (CAAD), Università del Piemonte Orientale “Amedeo Avogadro”, 28100 Novara, Italy; simonarevello@hotmail.com; 3 Mesothelioma, Melanoma and Rare Cancer Unit, Azienda Ospedaliera Universitaria SS Antonio e Biagio e Cesare Arrigo, 15121 Alessandria, Italy; 4 Independent Researcher, 28100 Novara, Italy

**Keywords:** pleural mesothelioma, immunotherapy, predictive biomarkers, tumor microenvironment, immune infiltrate, epigenetic modulators, vaccine, oncolytic virus, cell therapies, microbiota

## Abstract

Diffuse pleural mesothelioma (PM) is a rare thoracic malignancy with historically limited treatment options and poor outcomes. Despite the recent breakthrough of dual immune checkpoint blockade (ICB)—notably the combination of anti-PD-1 and anti-CTLA-4 therapies—clinical responses remain variable and overall survival gains modest. Consequently, there is an urgent need for multidimensional biomarkers and adaptive trial designs to unravel the complexity of PM immune biology. This review provides a comprehensive overview of current evidence on how histological subtypes (epithelioid vs. non-epithelioid) influence ICB efficacy, highlighting distinct genetic landscapes (e.g., BAP1, CDKN2A, NF2 mutations) and tumor microenvironment (TME) features, including immune infiltration patterns and PD-L1 or VISTA expression, that underlie differential responses. We further examine intrinsic tumor factors—such as mutational burden and checkpoint ligand expression—and extrinsic determinants, including immune cell composition, stromal architecture, patient immune status, and microbiota, as modulators of immunotherapy outcomes. We also discuss the rationale behind emerging strategies designed to enhance ICB efficacy, currently under clinical evaluation. These include combination regimens with chemotherapy, radiotherapy, surgery, epigenetic modulators, anti-angiogenic agents, and novel immunotherapies such as next-generation checkpoint inhibitors (LAG-3, VISTA), immune-suppressive cell–targeting agents, vaccines, cell-based therapies, and oncolytic viruses. Collectively, these advancements underscore the importance of integrating histological classification with molecular and microenvironmental profiling to refine patient selection and guide the development of combination strategies aimed at transforming “cold” mesotheliomas into “hot,” immune-responsive tumors, thereby enhancing the efficacy of ICB.

## Simple Summary

Pleural mesothelioma is an aggressive cancer with few effective treatments and poor survival for most patients. Although dual immune checkpoint blockade has recently emerged as a valuable therapeutic option, only a minority of patients benefit, and the reasons for this variability remain unclear. This review examines how the genetic and immune characteristics of different mesothelioma subtypes influence their response to immunotherapy. By integrating tumor-intrinsic determinants with features of the tumor microenvironment, environmental influences, and the patient’s immune status, we aim to clarify the factors driving sensitivity or resistance to immune checkpoint blockade. We also discuss new therapeutic strategies designed to render resistant tumors more responsive to immune attack. A deeper understanding of these mechanisms may guide the development of more effective, personalized treatment combinations and ultimately improve outcomes for patients with this challenging disease.

## 1. Introduction

Diffuse pleural mesothelioma (PM) is an aggressive malignant tumor of the pleural lining strongly linked to asbestos exposure [1]. Despite advances in oncology, it remains a disease with poor prognosis, with a median survival around 12 months for advanced disease [2]. For decades, cisplatin plus pemetrexed chemotherapy represented the standard first-line regimen, providing only modest survival benefits. PM comprises three main histologic subtypes (i.e., epithelioid, sarcomatoid, and biphasic), differing in prevalence, molecular features, and clinical behavior. Epithelioid PM, which accounts for about 60–70% of cases, generally confers a more favorable prognosis than the sarcomatoid (10–20%) and biphasic subtypes [3]. This histologic heterogeneity holds both prognostic and therapeutic implications.

In recent years, advances in tumor immunology have reshaped mesothelioma treatment. Most notably, immune checkpoint blockades (ICBs) targeting PD-1/PD-L1 and CTLA-4 have shown clinical activity in mesothelioma, leading to the approval of nivolumab (NIVO) (anti-PD-1) plus ipilimumab (IPI) (anti-CTLA-4) as a first-line option for unresectable PM [4]. Biologically, the PD-1/PD-L1 axis primarily acts in peripheral tissues, including the tumor microenvironment (TME), to inhibit effector T-cell activity and limit anti-tumor immune responses [5]. In contrast, CTLA-4 regulates an earlier phase of T-cell activation in secondary lymphoid organs by out-competing the costimulatory receptor CD28 for B7 ligands (CD80/CD86), thereby dampening the priming of naïve T-cells [6]. Because these mechanisms are non-redundant, their combined blockade provides a strong rationale for synergistic anti-tumor immunity. While both CTLA-4 and PD-1 blockade were first shown to be effective in advanced melanoma, subsequent clinical experience has demonstrated that PD-1/PD-L1 inhibition is generally safer and more effective than CTLA-4 blockade when used as monotherapy [7]. Consequently, PD-1/PD-L1 inhibitors have become the backbone of immunotherapy across multiple cancer types, whereas CTLA-4 inhibitors are now most commonly used in combination regimens, where the benefit of synergistic immune activation outweighs the toxicity limitations, as exemplified by the NIVO + IPI regimen for unresectable PM. This milestone represented the first systemic therapy approval in over 15 years and signaled the beginning of the “immunotherapy revolution” in mesothelioma management [8].

Despite this breakthrough, responses to ICB still remain inconsistent. Approximately 20% of PM patients derive no benefit (primary refractory disease) and a substantial fraction show only transient responses [4]. Notably, emerging evidence from clinical trials suggests that histologic subtype influences ICB outcomes. Patients with non-epithelioid (sarcomatoid or biphasic) mesothelioma seem to gain greater benefit from ICB than those with epithelioid histology [9]. Although this difference may simply reflect the long-recognized chemoresistance of sarcomatoid mesotheliomas, it could also stem from underlying biological disparities, such as genetics and tumor immune microenvironment (TME), between subtypes.

PM is generally considered an “immune-cold” or poorly immunogenic tumor, as single-agent checkpoint inhibitors give rise to lower response rates in PM than in highly immunogenic cancers like melanoma or lung cancer [8]. The tumor microenvironment (TME) of PM s typically enriched with immunosuppressive cells (e.g., M2 macrophages) and characterized by a dense fibrotic stroma that may restrict the infiltration of effector T cells [10]. Tumor cells commonly express inhibitory ligands such as PD-L1 and VISTA [11], which further dampen antitumor immunity. These combined factors contribute to immune evasion and reduced the efficacy of ICBs, resembling mechanisms observed in other refractory malignancies, including pancreatic ductal adenocarcinoma (PDAC) or glioblastoma (GMB), which share features of low T-cell infiltration and immune suppression.

Given these challenges, there is an urgent need to delineate the determinants of response and resistance to ICBs in PM. Identifying predictive biomarkers and understanding the interplay between tumor-intrinsic features (e.g., mutations and neoantigens) and tumor-extrinsic immune context (e.g., density of cytotoxic T and B cells vs. immunosuppressive T and myeloid cells) may help identify biomarkers to guide treatment selection and combination strategies.

This review provides a comprehensive synthesis of current evidence on predictors of ICB response in PM, integrating clinical, molecular, and immunologic perspectives. It contrasts epithelioid and non-epithelioid subtypes in terms of genomic alterations, TME characteristics, and therapeutic outcomes, highlighting how these biological distinctions shape differential sensitivity to ICB. In addition, it examines ongoing clinical trials and emerging combinatorial strategies aimed at enhancing immunotherapy efficacy and durability By bridging clinical observations with mechanistic insights, this review aims to provide a roadmap for the rational optimization of ICB in mesothelioma, moving toward precision immuno-oncology for this deadly disease.

## 2. Methodology

We conducted a narrative, scope-restricted review on determinants of immune checkpoint inhibitor response in pleural mesothelioma. Searches ran in PubMed/MEDLINE, Scopus, Web of Science Core Collection, and ClinicalTrials.gov for records dated 1 January 2020–9 November 2025. The strategy combined disease and histology terms (epithelioid, biphasic, sarcomatoid) with therapy, biomarker, and microenvironment descriptors (PD-1/PD-L1/CTLA-4 agents; BAP1, NF2, LATS2, CDKN2A, SETD2, TP53; tumor mutational burden, HLA, interferon (IFN) signaling, STING; macrophage polarization, fibroblasts, immune exclusion, angiogenesis, hypoxia). We harmonized syntax to each database and expanded the corpus through backward and forward citation chaining from key trials and translational studies. An AI-assisted deep search supported synonym expansion, query normalization, batch export, and duplicate detection; generative tools were restricted to retrieval logistics and language editing. Authors verified bibliographic identity, re-checked key numerical values against source PDFs, and made all inclusion and exclusion decisions to ensure data integrity and prevent factual or interpretative errors.

Screening occurred in two stages by two authors. Title and abstract screening applied English language, pleural focus, ICI relevance, and date window. Full texts were then assessed for histology-resolved outcomes, genomic or microenvironment correlates of response or resistance, including spatial profiling, multiplex IHC/IF, single-cell or bulk RNA-seq, or interventional combinations relevant to ICI. We excluded non-pleural mesothelioma without pleural-specific data, non-English items, pre-2020 items unless foundational, editorial material without original data, and preprints not peer-reviewed. Where only an abstract was accessible, the study was flagged and listed in an access-limited section after the references. Finally, to mitigate bias inherent in narrative designs, we prioritized prospective cohorts and histology-resolved analyses when available, triangulated biomarker associations across independent datasets or orthogonal platforms, and highlighted areas where subgroup sizes limited inference. Authors performed all inclusion/exclusion decisions, full-text evaluations, data extraction, and interpretation; AI support remained restricted to search logistics and language editing.

## 3. Current Clinical Efficacy of ICB in PM

ICB has dramatically changed the treatment landscape of PM in recent years. Early trials of single-agent ICBs in relapsed PM showed signs of activity but modest overall success (Table 1). For example, the KEYNOTE-028 trial reported objective response rates (ORRs) of ~20% in PD-L1-positive mesothelioma patients treated with the PD-1 inhibitor pembrolizumab (PEMBRO) [12], whereas the KEYNOTE-158 study found ORRs of ~8–20% in unselected, previously treated populations [13]. Similarly, nivolumab (NIVO) monotherapy achieved a ~29% ORR in the phase II MERIT trial in Japan [14], while the randomized phase III CONFIRM trial revealed that NIVO significantly improved progression-free survival (PFS) and overall survival (OS) compared with placebo in previously treated patients with PM [15]. In this trial, the median OS reached 10.2 months with NIVO vs. 6.9 months with placebo (hazard ratio [HR] ~0.69) [15], establishing single-agent anti-PD-1 therapy as an effective option after chemotherapy failure. It is important to point out, however, that most of patients enrolled in the aforementioned trials had a PM with epithelioid histology, the predominant subtype, and a survival benefit was observed even within this less immunogenic subgroup.

Dual immune checkpoint inhibition has shown an even more favorable clinical outcome. The phase II IFCT-1501 MAPS2 trial compared NIVO monotherapy with the combination of NIVO and ipilimumab (IPI) in patients progressing after platinum/pemetrexed chemotherapy [9]. Both arms met the primary endpoint of 12-week disease control (~44–50%), but the NIVO + IPI combo arm achieved a higher response rate (28% vs. 19%) and longer median OS (15.9 vs. 11.9 months), albeit with increased toxicity. These findings, validated by subsequent studies, supported the use of dual ICB as a promising strategy for relapsed PM, especially given the scarcity of effective second-line options.

A major breakthrough occurred in the first-line setting with the phase III CheckMate 743 trial, which compared first-line NIVO + IPI to standard chemotherapy in unresectable PM [4]. The results were practice-changing: median OS was 18.1 months with NIVO + IPI vs. 14.1 months with chemo (HR 0.74, *p* = 0.002). At 2 years, 41% of patients receiving immunotherapy were alive compared with 27% in the chemotherapy arm, translating to a 26% reduction in mortality risk. These results resulted in regulatory approvals of the dual ICB regimen by the FDA in October 2020 and by the EMA in July 2022 [4]. Interestingly, although response rates and median PFS were comparable between immunotherapy and chemotherapy (~40% vs. 43% and ~6.8 vs. 7.2 months, respectively), responses with ICB were substantially more durable (median duration of response 11.0 vs. 6.7 months). At 3-year follow-up, ~23% of patients on NIVO + IPI were still alive vs. ~15% of those receiving chemotherapy, with 28% of responders to ICB maintaining sustained responses [16]. Importantly, disease-related symptoms and health-related quality of life (HRQoL) were significantly improved in the ICB arm throughout the 30-month follow-up [9]. Notably, the OS benefit was achieved without an increase in grade 3–4 toxicity (~30% in both arms), though immune-related adverse effects differed qualitatively from chemotherapy toxicities [4].

Subsequent real-world studies examined the efficacy and safety of dual-ICB outside clinical trial settings. Multicenter retrospective cohorts from France (201 patients) [17] and Latin America (96 patients) [18] confirmed both the survival and safety profiles reported in CheckMate 743. Conversely, a Swiss cohort study including 47 first-line and 62 later-line ICB-treated patients reported less favorable survival outcomes [19,20]. Analyses of Australian cohorts, each comprising roughly 100 patients, highlighted either an equal efficacy of dual ICB compared to chemotherapy [21] or a reduced survival outcome along with higher toxicity [20]. Similarly, a prospective Dutch cohort of 184 patients showed higher rates of treatment-related adverse events (TRAEs) but comparable efficacy [22]. Thus, in contrast to the CheckMate 743 trial, real-world data have raised questions regarding the magnitude and consistency of immunotherapy benefit across the broader mesothelioma population. Variations in patient selection, histologic subtype, comorbidities, and treatment sequencing likely contributed to these discrepancies. Nonetheless, when analyzed by histology, real-world studies consistently corroborated the CheckMate 743 observation of pronounced benefit with dual ICB in non-epithelioid tumors.

In CheckMate 743, patients with sarcomatoid or biphasic mesothelioma receiving NIVO + IPI had a median OS of 18.1 months vs. 8.8 months with chemotherapy (HR ~ 0.46). In contrast, epithelioid mesothelioma patients showed a more modest benefit: their median OS was 18.7 months with ICB vs. 16.5 months with chemotherapy (HR ~ 0.86) [4]. Further underscoring this disparity, three-year survival rates were 22% (ICB) vs. 4% (chemo) in non-epithelioid patients and 24% vs. 19% in epithelioid ones [16]. As a result, current clinical guidelines strongly recommend the combination of NIVO + IPI as a frontline treatment for non-epithelioid mesothelioma [23]. In contrast, the superior efficacy of dual ICB therapy for the epithelioid subtype remains debated [24], underscoring the urgent need for predictive biomarkers to guide clinical decision making.

Current understanding of the biological determinants underlying the differential ICB responsiveness between epithelioid and non-epithelioid tumors, as well as ICB efficacy across histological subtypes, is discussed in the subsequent sections.

**Table 1:** Clinical trials of immune checkpoint inhibitors in PM.

Trial	Phase	Therapy	Histotypes *	ORR	Median PFS (mos)	Median OS/Outcome (mos)	Ref
Check-Mate 743	III	Nivolumab + Ipilimumab vs. Platinum/Pemetrexed	Epithelioid ~75%, Non-epithe- lioid ~25%	38% vs. 43%	6.8 mos vs. 7.2 mos	18.1 mos vs. 14.1 mos (HR 0.74); higher in non-epithelioid (HR 0.46)	[4]
DREAM	II	Durvalumab + Platinum/Pemetrexed (single-arm)	Epithelioid 77% Biphasic 13%, Sarcomatoid 10%	48%	6.9 mos	18.4 mos (12-mos OS 70%)	[25]
PrE0505	II	Durvalumab + Platinum/Pemetrexed (single-arm)	Epithelioid 79% Biphasic 12%, Sarcomatoid 9%	56%	6.7 mos	20.4 mos	[26]
CONFIRM	III	Nivolumab vs. Placebo (post-chemo)	Epithelioid 85%, Non-epithelioid 15%	4% vs. 0%	3.0 mos vs. 1.8 mos	10.2 mos vs. 6.9 mos (HR 0.69)	[9]
MERIT	II	Nivolumab (single-arm, Japan)	Epithelioid 89% Non-epithelioid 11%	29%	6.1 mos	17.3 mos	[14]
MAPS2	II	Nivolumab ± Ipilimumab (post-chemo)	Epithelioid 79% Biphasic 15% Sarcomatoid 6%	28%/19%	4.7 mos/2.6 mos	15.9 mos/11.9 mos	[9]

* Percentages reported for histological PM subtypes correspond to the proportion of enrolled patients in each study.

## 4. Epithelioid vs. Non-Epithelioid Pleural Mesothelioma: Potential Genetic and Immune Drivers of Divergent Prognoses and Therapeutic Outcomes

The histologic subtypes of PM are not merely morphological categories; they reflect distinct molecular profiles and TME, which influence disease behavior and therapy response. Epithelioid mesothelioma, the most prevalent subtype, is characterized by cohesive polygonal tumor cells that form tubulopapillary or solid structures, often embedded in a dense stromal matrix [27]. This variant typically carries a more favorable prognosis, with median survivals exceeding 18 months in recent series, when treated aggressively [8]. In contrast, sarcomatoid mesothelioma consists of spindle-shaped cells resembling fibrosarcoma and is the most aggressive subtype, with a median survival as short as ~4–8 months if untreated or treated with chemotherapy alone [8,23,28,29]. However, the introduction of dual ICB regimens has substantially altered this outlook, extending OS by more than 10 months [4], and narrowing the prognostic gap between histologic subtypes. Biphasic mesothelioma, comprising both epithelioid and sarcomatoid components, shows an intermediate clinical course that aligns more closely with the dominant histologic component.

The 2021 WHO classification has further refined these categories by introducing grading for epithelioid tumors based on architectural patterns (e.g., solid vs. micropapillary) and nuclear atypia, both of which strongly correlate with clinical outcome [28]. Parallel advancements in molecular profiling have provided additional insight into mesothelioma heterogeneity. In particular, large-scale transcriptomics studies have revealed four primary subgroups: epithelioid (E), sarcomatoid (S), and two biphasic variants (biphasic-E/S) [30]. Subsequent histo-molecular characterization using E- and S-scores further evidenced tumor heterogeneity [31].

These molecular refinements bear direct clinical implications as histology serves not only as a key prognostic factor in PM, but also as a predictive biomarker for treatment selection. The striking efficacy difference in NIVO + IPI-based immunotherapy vs. cisplatin + pemetrexed-based chemotherapy for sarcomatoid PM [4], in contrast with the slight benefit observed in epithelioid tumors, underscores histology-based stratification as critical for clinical decision-making. Whether the superior outcomes with dual ICB in non-epithelioid PM reflect a true enhanced immunotherapy sensitivity or the relative chemoresistance of these tumors remains an area of active investigation. Nonetheless, converging genomic, transcriptomic, and immunologic data (Table 2) [30,31,32,33] indicate that divergent immune microenvironments and genetic drivers underlie these differential therapeutic outcomes, providing key mechanistic insight into subtype-specific responsiveness to ICBs.

Genetically, all PM subtypes share common driver alterations that result in the inactivation of tumor suppressor genes. Specifically, the majority of PMs (~50–70%) harbor homozygous deletions on chromosomes 9p21, which lead to the loss of *CDKN2A* (p16), often associated with the co-loss of *CDKN2B* (p15) and *MTAP*. In parallel, ~40–60% of cases display loss-of-function mutations in BRCA1-associated protein 1 (*BAP1*) on chromosome 3p21 [36]. Other frequently altered genes include *NF2* (~30–40% of cases), encoding merlin, a key regulator of the Hippo signaling pathway, and less frequently *TP53* (~10–20%), *LATS2*, and other tumor suppressors involved in cell-cycle control and contact inhibition [36,43].

Interestingly, the distribution of certain mutations differs by histology [34]. In particular, epithelioid mesotheliomas exhibit the highest prevalence of BAP1 mutations, which is reported in up to 60% of cases [34]. In contrast, pure sarcomatoid tumors tend to display lower rates of BAP1 loss (20–30%) [29], although one sarcomatoid PM series revealed an unusually high frequency exceeding 90% [44]. In good agreement with the higher prevalence of BAP1 loss in epithelioid mesothelioma, germline BAP1 mutations, characterizing BAP1 tumor predisposition syndrome, typically give rise to epithelioid mesotheliomas, which paradoxically appear to have a less aggressive behavior and a prolonged median survival (~5–7 years) [45,46].

Homozygous deletions of *CDKN2A*, which often co-occur with *BAP1* loss [11], is reported in both subtypes. However, consistent with the higher rate of BAP1 alterations in epithelioid tumors, some studies indicate that *CDKN2A* loss is more uniformly distributed in epithelioid PM as well [47]. Conversely, another study comparing the molecular profiles of 105 non-epithelioid vs. 235 epithelioid PMs highlighted a significantly higher frequency of alterations involving *CDKN2A* (68% vs. 44%), *CDKN2B* (59% vs. 38%), *MTAP* (51% vs. 29%), *NF2* (50% vs. 30%), and *TERT* (19% vs. 5%) in the non-epithelioid group [34]. Although it was observed in only 10–15% of PM overall, *TP53* mutations also appear to cluster in non-epithelioid cases and to be associated with particularly poor prognosis [30]. The tumor suppressor *NF2* (merlin), which has emerged as a late event in the progression of PM [37,48] is frequently mutated across all subtypes, but some studies suggest a slightly higher prevalence in non-epithelioid tumors, aligning with the observation that Hippo pathway disruption can drive a more aggressive mesenchymal phenotype [37,38].

In summary, epithelioid PM is often defined by *BAP1* loss, along with high nuclear p16 expression loss and lower incidence of TP53 alteration, whereas sarcomatoid PM more frequently shows *CDKN2A/2B* (p16/p15) deletion with *MTAP* co-deletion, *NF2* loss, and can harbor *TP53* mutations [36,43]. These genetic differences may critically impact immunogenicity and treatment susceptibility. For example, by modulating DNA damage repair and apoptosis, BAP1 loss may enhance susceptibility to synthetic lethality-based strategies [49]. Furthermore, according to preclinical studies, *BAP1* loss is associated with an inflammatory tumor phenotype, potentially acting as a favorable biomarker for immunotherapy responsiveness [35,36]. Conversely, *CDKN2A/2B* loss correlates with resistance to PD-1 blockade in preclinical models [36], suggesting that CDK4/6 inhibitors, either alone or combined with ICB, may offer therapeutic benefit [15,50]. In addition, while specific genetic alterations may influence PM sensitivity to various systemic therapies, co-mutation patterns, such as *CDKN2A* loss with *BAP1* or *NF2* co-loss, appear to define distinct molecular subgroups that could display differential sensitivity to therapies [43].

Progress in characterizing PM heterogeneity through integrative multi-omics approaches has provided valuable insight into the cross-talk between genomic and immune determinants of tumor behavior. Building upon earlier immune profiling studies that delineated TME heterogeneity across histological subtypes [30], recent analyses combining genomics, epigenomics, and transcriptomics have uncovered the existence of a dynamic interplay between the immune landscape and tumor cell-intrinsic genomic and epigenetic alterations [33,36,51]. A recent study integrating TME characterization via imaging mass cytometry (IMC) with whole genome sequencing data reported an inverse correlation between macrophage infiltration, CD4^+^ T-cell abundance, and tumor mutational burden (TMB). Interestingly, this phenogenomic feature correlated with improved outcomes in a predominantly epithelioid PM cohort (n = 22) treated with chemotherapy [52], raising questions about its predictive role in non-epithelioid PM and other treatment settings.

Immune profiling further refines our understanding of PM biology, revealing subtype-specific immune architectures. Epithelioid mesotheliomas tend to exhibit an immune-excluded phenotype with an abundance of tumor-infiltrating lymphocytes (TILs) in the stromal areas—particularly CD4^+^ Th1-polarized T cells and B cells—but with fewer T cells making direct contact with tumor islets [8,41]. Furthermore, transcriptional profiling studies, including single-cell RNA sequencing (scRNA-seq) analysis, showed a higher presence of natural killer (NK) cells in epithelioid subtype [31,33], suggesting NKG2A:HLA-E interaction between NK and tumor cells as a novel, important therapeutic axis in epithelioid PM [42]. In contrast, sarcomatoid mesotheliomas often display an immune-infiltrated yet immunosuppressive phenotype, characterized by abundant TILs, M2-polarized tumor-associated macrophages (TAMs), and T regulatory cells (Tregs) [8,31]. Moreover, transcriptional profiling studies revealed additional enrichment of fibroblasts, endothelial cells, TILs, and TAMs, implying that the immune infiltrate within the sarcomatoid component supports angiogenesis [31]. Consistently, a sarcomatoid signature, including a novel fetal-like, endothelial cell population, along with CXCL9^+^ macrophages and TILs (e.g., cytotoxic and exhausted T cells, and Tregs), has been recently defined by scRNA-seq analysis and subsequently validated using imaging and bulk deconvolution analyses across multiple independent cohorts [42]. In agreement with immune infiltration patterns linked to S- and E-scores [31], Torricelli et al. used spatially resolved, high-dimensional transcriptomics to compare epithelioid (E) and sarcomatoid (S) regions within biphasic PM, uncovering extensive remodeling of the tumor microenvironment during the epithelioid-to-sarcomatoid transition [52]. This study also demonstrated a widespread enrichment of immune cell populations, encompassing both myeloid and lymphoid lineages, along with increased fibroblast and endothelial cell counts in the sarcomatoid compartment. Noteworthy, progression toward the sarcomatoid phenotype was associated with elevated TGF-β1 levels, M2-polarized TAMs, and markers of T-cell exhaustion. Collectively, these findings suggest that TGF-β1-polarized TAMs promote an immune suppressive microenvironment, highlighting their potential as therapeutic targets in sarcomatoid PM [52].

Another striking difference between epithelioid and non-epithelioid mesotheliomas concerns immune checkpoint ligand expression. PD-L1 is markedly upregulated in non-epithelioid mesotheliomas, where its association with S-score and TIL exhaustion signatures supports the rationale for dual ICB [31]. Consistent with transcriptomics data, immunohistochemical analyses have shown that sarcomatoid and biphasic tumors are 4–5 times more likely to express PD-L1 than epithelioid tumors [39]. For instance, one study reported PD-L1 staining of at least 1% in ~40–50% of sarcomatoid/biphasic PM compared with ~10–15% of epithelioid cases. When a higher threshold of 5 percent was applied, PD-L1 positivity was observed in roughly 25–30 percent of sarcomatoid and biphasic tumors, but in fewer than 5 percent of epithelioid tumors [40]. Furthermore, high PD-L1 expression in PM appears to correlate with poor survival [39], largely reflecting the aggressive nature of these histotypes. In contrast, VISTA, an alternative immune checkpoint receptor, is preferentially expressed in epithelioid mesotheliomas [31,33] where it may mediate immune evasion in these typically PD-L1-low tumors [11].

**Figure 1:**
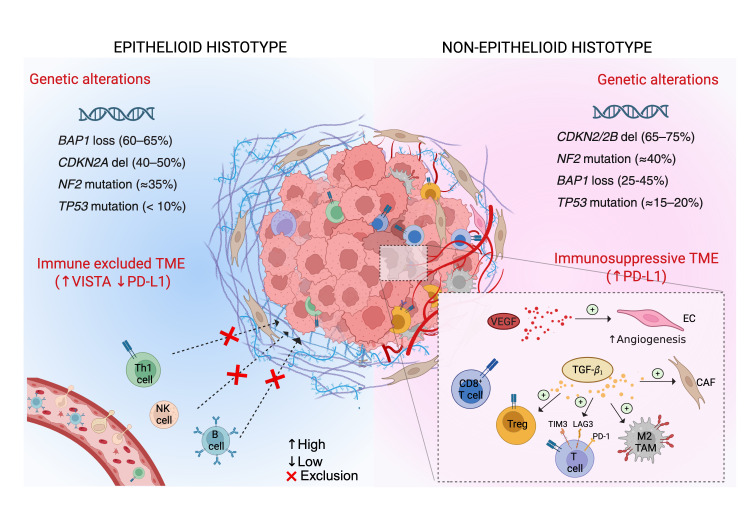
Intrinsic (genetic) and extrinsic (microenvironmental) factors vary across histologic subtypes and influence response to immune checkpoint blockade (ICB) therapy. The tumor microenvironment (TME) of epithelioid pleural mesothelioma (PM) is typically immune-excluded, whereas non-epithelioid PM displays an inflamed but immunosuppressive phenotype. These distinctions provide a rationale for histology-tailored immunotherapeutic strategies. Th1 cell, T helper 1 cell; NK, Natural killer cell; TAM, tumor-associated macrophage; Treg, T regulatory cell; TGF-b1, Transforming growth factor-b1; VEGF, vascular endothelial growth factor; EC, endothelial cell; CAF, cancer-associated fibroblast. The figure was created with BioRender (www.BioRender.com, accessed on 12 November 2025).

Taken together, these genomic and immunologic insights delineate a clear dichotomy between epithelioid and non-epithelioid PM. While sarcomatoid tumors demonstrate greater responsiveness to PD-1/PD-L1 inhibition, likely due to their pre-existing CD8^+^ T cell infiltrates and PD-L1 expression, epithelioid tumors may require combinatorial approaches targeting VISTA or enhancing T-cell recruitment to overcome immune resistance. Understanding these subtype-specific immunogenomic interactions is expected to provide a robust framework for precision immunotherapy and the rational design of biomarker-driven clinical trials (Figure 1).

## 5. Intrinsic and Extrinsic Determinants of ICB Response

Outside histological classification, the variable sensitivity of mesothelioma to dual ICB, especially among epithelioid tumors, likely reflects the extensive molecular diversity observed both within and across tumors [27,28,49]. This clinical and molecular heterogeneity underscores the need to identify tumor-intrinsic properties and microenvironmental factors that modulate ICB responsiveness, even among cases with identical histologic features. Current research aims to uncover these determinants to improve patient stratification and optimize treatment outcomes. In the following section, we review promising biomarkers from recent clinical trials and emerging molecular candidates in PM, regardless of histological subtype.

### 5.1. Neoantigens: Tumor Mutational Burden and Chromosomal Rearrangements

**Figure 2:**
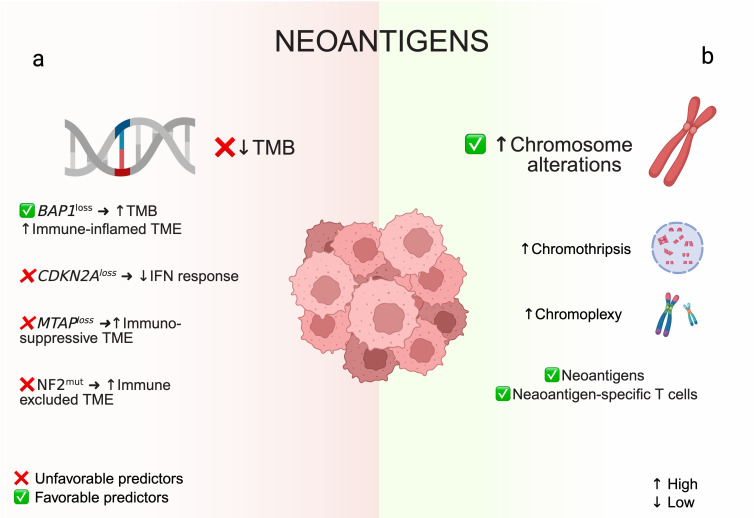
Contribution of TMB and chromosomal alterations to neoantigen generation. (**a**) Although PM displays a relatively low TMB compared with other ICB-responsive cancers, BAP1 mutations are associated with both increased TMB and a more inflamed TME, suggesting enhanced sensitivity to ICB. In contrast, CDKN2A, MTAP, and NF2 alterations correlate with immunosuppressive or immune-excluded phenotypes, contributing to ICB resistance. (**b**) PM is characterized by extensive chromosomal rearrangements, including chromothripsis and chromoplexy, which can generate novel and potentially immunogenic neoantigens capable of driving tumor-specific T-cell responses and ICB sensitivity. The figure was created with BioRender (www.BioRender.com, accessed on 9 December 2025).

Tumors with high TMB generally produce a greater number of neoantigens, thus enhancing immunogenicity. Fittingly, high TMB is recognized as a predictive biomarker for ICB responsiveness in several malignancies, including non-small cell lung cancer (NSCLC), melanoma, and other advanced solid tumors, irrespective of cancer type [53]. All the histological subtypes of PM typically have moderate TMB—lower than smoking-associated lung cancers or melanoma, but higher than pediatric or virus-driven tumors. With a median of 1 to 2 mutations/Mb (total somatic mutations on the order of 50–200 per exome) [34], PM is at the lower end of the spectrum of ICB-responsive cancers (Figure 2a). This biological feature aligns with the modest ORRs of single-agent ICBs (~10–20%) observed in clinical studies.

Nevertheless, TMB is not uniform across mesothelioma. A subset of patients with impaired DNA repair mechanisms—such as those carrying germline BAP1 mutations or other genetic predispositions—tend to accumulate more mutations, resulting in higher TMB levels and, in some reports, improved survival following immunotherapy [26]. On the other hand, the CheckMate 743 study found no clear correlation between TMB and benefit from NIVO + IPI [16]. Unlike NSCLC or melanoma, mesothelioma patients with high TMB did not demonstrate a significant survival benefit from ICB compared to TMB-low cases. This implies that TMB alone poorly predicts immunogenicity in PM, possibly because even TMB-high cases fall in an intermediate mutational range, or because dominant immunosuppressive mechanisms within the TME counteract this effect.

Besides single-nucleotide variants, structural chromosomal rearrangements represent another defining genomic hallmark of PM across subtypes (Figure 2b) [54]. Large-scale deletions/insertions, chromoplexy, and chromothripsis can generate novel gene fusions and neoepitopes presented on MHC molecules, thereby triggering clonal expansion of tumor-infiltrating T cells. Furthermore, whole-genome sequencing, including mate-pair analyses, has linked such chromosomal rearrangements to both neoantigen formation and the presence of circulating neoantigen-specific T cells in PM [54]. Thus, in light of this evidence, antigen quality rather than quantity may more accurately reflect the immunogenic potential of mesothelioma.

In support of this concept, PM frequently expresses immunogenic tumor-associated antigens, such as mesothelin and Wilms tumor 1 (WT-1), both capable of eliciting specific immune responses. This suggests that neoantigen immunogenicity may outweigh TMB as a predictive biomarker for ICB efficacy. The PrE0505 trial further reinforced this hypothesis by reporting improved clinical outcomes with chemo-immunotherapy in a subset of mesotheliomas exhibiting both high clonal neoantigen burden and diverse T-cell receptor repertoires [26].

Together, these data indicate that the interplay between genomic instability, neoantigen landscape, and T-cell diversity is central to defining the immunogenic potential and therapeutic responsiveness of mesothelioma.

### 5.2. PD-L1 and Other Inhibitory Immune Checkpoints

PD-L1 expression, whose correlation with survival outcomes has led to its FDA approval as a predictive biomarker for ICB efficacy for different types of cancers [55], seems to have limited predictive value in PM. Indeed, in the CheckMate 743 trial, PD-L1 expression (≥1% vs. <1%) did not significantly discriminate which patients benefited from ICB, but unexpectedly influenced chemotherapy outcomes [16]. Specifically, PD-L1-negative tumors showed superior chemotherapy responses compared to PD-L1-positive cases, implying that PD-L1 status may help identify chemotherapy-resistant subgroups [16]. Similarly, the KEYNOTE-483 trial demonstrated that the addition of pembrolizumab to chemotherapy provided benefit regardless of PD-L1 expression [56]. Earlier studies in pretreated cohorts, including PROMISE-meso, KEYNOTE-158, MERIT, and CONFIRM, also reported no correlation between PD-L1 expression and clinical response to ICB [57]. However, these findings should be interpreted with caution since PD-L1 assessment was primarily based on archival diagnostic biopsies, which may not reflect expression dynamics during disease progression. A more reliable evaluation of PD-L1 as a predictive biomarker for ICB would require testing in treatment-naïve patients receiving frontline immunotherapy. Additionally, most studies focused on tumor cells only, neglecting their role within the immunosuppressive TME, particularly in TAMs. Therefore, assessing either PD-L1 or PD-1 in both tumor and stromal cells could provide a more accurate prediction of ICB responsiveness. In this regard, a study on pre-treated PM patients found that PD-1-expressing CD8^+^ T cells and CD68^+^ macrophages, but not PD-L1-expressing tumor or immune cells, were independent predictors of response to pembrolizumab [58].

In this context, assessing additional immune checkpoints, such as VISTA, LAG-3, and TIM-3, may enhance patient stratification by revealing immune responses that are suppressed but still capable of reactivation. Furthermore, the integration of markers of T-cell exhaustion through the analysis of these inhibitory checkpoints could enable a more accurate prediction of ICB benefit. For example, high VISTA expression, found mainly in tumor-infiltrating immune cells and, to a lesser extent, in tumor cells [31,33], may contribute to intrinsic ICB resistance by providing alternative inhibitory signals to T cells. Consistently, in a small cohort of PM patients receiving the combination of anti-PD-L1 (atezolizumab) and anti-vascular endothelial growth factor (VEGF) (bevacizumab) in the second-line setting, non-responders showed an enrichment of VISTA-expressing CD68^+^ macrophages [32]. This observation provides a rationale for ongoing early-phase clinical trials of anti-VISTA antibodies, which may be particularly relevant for tumors with low PD-L1 but high VISTA expression. In parallel, other inhibitory receptors such as LAG-3 and TIM-3 are frequently upregulated on TILs in mesothelioma, reflecting an exhausted immune state [59]. Of note, LAG-3, together with CD8A, STAT1, and CD274/PD-L1, forms part of an exploratory four-gene inflammatory signature associated with improved survival in patients receiving immunotherapy [16]. Congruently, tumors showcasing an “inflamed” transcriptional profile, characterized by elevated IFN-γ signaling and T-cell activation, showed greater clinical benefit from NIVO + IPI therapy [16].

**Figure 3:**
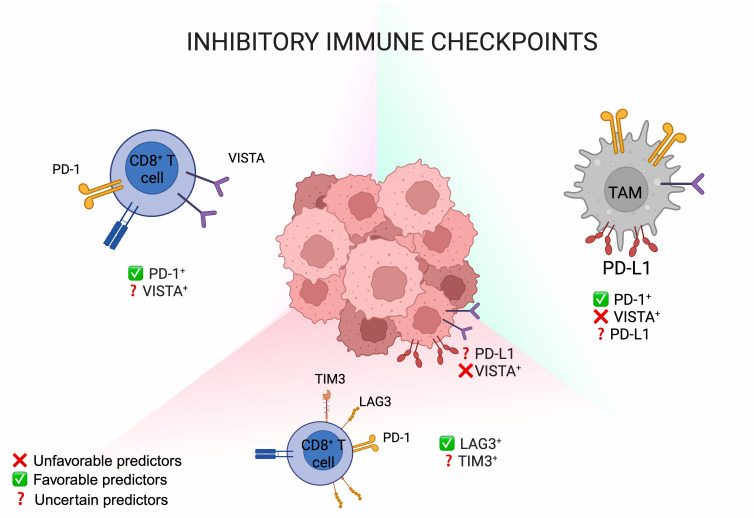
Inhibitory immune checkpoints as predictive biomarkers for ICB efficacy in PM. Current evidence indicates that PD-L1 expression on tumor cells or TAMs, as well as VISTA and TIM-3 expression on CD8^+^ T cells, have limited predictive value for ICB response in PM. VISTA expression on tumor cells and TAMs is generally associated with poor outcomes, whereas PD-1 expression on CD8^+^ T cells and TAMs correlates with improved response to ICB. Finally, LAG-3 has emerged as part of an exploratory four-gene inflammatory signature linked to better survival in patients treated with immunotherapy. The figure was created with BioRender (www.BioRender.com, accessed on 9 December 2025).

Collectively, these findings indicate that PD-L1 expression alone is not an adequate criterion for patient selection in PM. A more reliable stratification strategy should incorporate comprehensive profiling of T-cell exhaustion markers (e.g., PD-1, CTLA-4, and LAG-3) to optimize patient selection for current dual ICB regimens (Figure 3) and guide the development of novel combination therapies targeting multiple immune pathways, particularly in tumors characterized by enhanced IFN-γ signaling and T-cell activation.

### 5.3. Tumor Suppressor Gene Alterations (BAP1, CDKN2A, NF2)

As discussed earlier, tumor suppressor gene alterations are ubiquitous in PM and play a significant role in shaping the immune response and therapeutic sensitivity (Figure 2a). Among these, BAP1-deficient mesotheliomas have drawn particular attention due to the pleiotropic regulatory functions of this gene, which range from DNA damage repair and cell cycle control to chromatin remodeling. Its loss may increase genomic instability, immunogenic cell death, and cytokine expression, potentially enhancing responsiveness to ICB [60]. Transcriptomic analyses from The Cancer Genome Atlas (TCGA) showed that BAP1 mRNA expression in mesotheliomas inversely correlates with a type I IFN gene signature [33,61]. This suggests that BAP1-deficient tumors have chronic IFN signaling—likely triggered by endogenous retroelement activation—which could simultaneously promote T-cell infiltration, exhaustion, and sensitivity to PD-1/PD-L1 blockade [62]. In good agreement, studies in both pleural and peritoneal mesothelioma have linked BAP1 deficiency to an inflamed TME, suggesting enhanced ICB sensitivity [35,63]. Specifically, loss of BAP1 was associated with reduced myeloid-derived suppressor cells (MDSCs) and increased inflammatory dendritic cells (DCs), macrophages, and exhausted T cells [35]. On the other hand, a study on a small clinical series of PM treated with ICB reported no significant survival differences based on BAP1 status [64]. While these conflicting results preclude firm conclusions, larger prospective studies are needed to definitively clarify the predictive value of BAP1.

Loss of *CDKN2A* is associated with a more aggressive, immunotherapy-resistant phenotype. Preclinical evidence indicates that p16 loss can lead to changes in cell cycle regulation and IFN signaling, leading to a PD-1-resistant tumor phenotype [36,50,65]. In addition, *MTAP* loss, which often accompanies *CDN2A* deletion, may support the generation of an immunosuppressive metabolic environment that dampens T-cell activity and ICB responsiveness. Indeed, accumulation of 5′-deoxy-5′-methylthioadenosine (MTA) in MTAP-deficient tumors suppresses human T-cell activation and effector function [66]. Fittingly, MTAP-deficient lung tumors show increased expression of exhaustion markers on T cells, reduced infiltration of T and NK cells, and accumulation of immunosuppressive cells (e.g., Tregs and MDSCs) [67].

NF2 mutations activate the Hippo/YAP signaling pathway, which has been shown to upregulate PD-L1 expression in tumors like melanoma and lung cancer [68,69], potentially making NF2-mutant tumors more immunoevasive. Accordingly, one study found a correlation between NF2 alterations and an immune-excluded phenotype in PM [11].

Overall, current findings indicate that tumor suppressor genotypes play a decisive role in determining the immune composition of the mesothelioma microenvironment. Thus, understanding how specific genomic alterations influence immune regulation constitutes a key step toward the identification of predictive biomarkers of ICB response to and the development of novel therapeutic strategies combining targeted and immune-based approaches.

### 5.4. Extrinsic Determinants: Tumor Microenvironment and Immune Contexture

**Figure 4:**
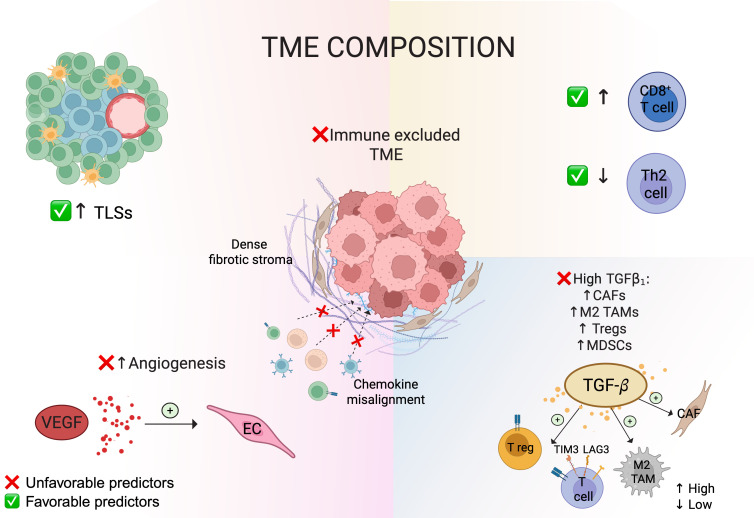
TME features influencing response to ICB in PM. The presence of TLS and a T-cell–inflamed phenotype, marked by abundant CD8^+^ T cells and reduced Th2-polarized CD4^+^ T cells, is associated with improved outcomes. In contrast, an immune-excluded TME—characterized by limited T-cell infiltration due to dense fibrotic stroma and chemokine misalignment—drives resistance to ICB. Additional resistance mechanisms include VEGF-mediated angiogenesis and heightened TGF-β signaling, which promote CAF expansion, M2 macrophage accumulation, increased Treg and MDSC levels, and impaired effector T-cell function. Collectively, these features shape the spatial and functional architecture of the TME, ultimately determining sensitivity or resistance to immunotherapy. The figure was created with BioRender (www.BioRender.com, accessed on 9 December 2025).

Similar to other cancer types, the extrinsic factors arguably play a leading role in determining the response of mesothelioma to ICB. These include the composition and functional orientation of immune cells, stromal architecture, and the presence of soluble mediators within the TME. Given the recent approval of ICB as a first-line therapy for PM, the predictive value of TME features for treatment response is under active investigation. Although definitive predictive markers are yet to be determined, preliminary insights can be drawn from our current understanding of TME dynamics in other malignancies, as well as from studies analyzing tissue biopsies and pleural effusion samples from chemotherapy-treated PM patients (Figure 4) [8,70,71].

In general, it is widely recognized that ICB responsiveness is associated with an immunologically “hot” TME, characterized by abundant CD8^+^ cytotoxic T cells (CTLs), Th1-polarized CD4^+^ T cells, a low ratio of immunosuppressive to effector cells (Tregs and MDSCs vs. CTLs and Th1 cells), and the presence of professional antigen-presenting cells, especially conventional DC1, capable of sustaining effective antigen presentation and T-cell priming [72,73]. In such tumors, effector T cells infiltrate deeply and establish direct contact with malignant cells, fostering immune-mediated cytotoxicity. In contrast, PM is typically considered an “immune-cold” tumor [8,70,71]. Many cases, especially certain epithelioid subtypes, exhibit an “immune desert” phenotype, where tumor nodules are embedded in a dense collagenous matrix with minimal T-cell infiltration. Others display an “immune-excluded” pattern, in which T cells accumulate at the periphery or within stromal regions but fail to penetrate tumor islets. Only a minority of cases present with an inflamed or “hot” phenotype, where T cells are interspersed with tumor cells. This phenotype is more frequently observed in sarcomatoid or high-grade epithelioid regions, which tend to harbor stronger inflammatory and immune signaling profiles.

A higher baseline of CD8 T-cell density in mesothelioma biopsies is consistently associated with better survival, establishing T-cell-inflamed tumors as a favorable prognostic biomarker, regardless of treatment modalities [8,70,71]. Integrative transcriptomics analyses by Alay A. et al. further refined this concept by identifying three distinct immune clusters (IG1-IG3) significantly associated with clinical outcomes [74]. Notably, the IG3 cluster—defined by a Th2^low^ cytotoxic T^high^ signature—not only predicted a better prognosis but also showed molecular features suggestive of enhanced responsiveness to ICB based on integrated gene expression and drug-response signatures [74].

Improved outcomes also seem to correlate with increased B lymphocyte infiltration [75,76] and the presence of tertiary lymphoid structures (TLSs), which are emerging as a predictive biomarker for ICB response across multiple cancer types [77]. Interestingly, the association between TLSs and prolonged survival in treatment-naïve epithelioid PM (n = 129) supports their potential use in stratifying ICB sensitivity within this histologically homogeneous subgroup.

An abundant population of TAMs with an immunosuppressive M2 phenotype is a hallmark of PM [78,79,80,81,82]. Although their overall prognostic value is being questioned [71], resistance to PD-1 blockade frequently coincides with the upregulation of macrophage- and stroma-related gene signatures, which contribute to immune exclusion and impair T-cell infiltration [83]. Congruently, in relapsed PM, high CD68^+^ macrophage density inversely correlated with CD45RO^+^CD8^+^ memory T-cell abundance and resistance to atezolizumab (anti-PD-L1) plus bevacizumab (anti-VEGF) therapy [32]. Preclinical studies further demonstrated that pharmacologic depletion of macrophages through CSF-1R inhibition enhances PD-1 blockade efficacy in sarcomatoid PM models [84]. Moreover, in a cohort of pre-treated patients, PD-1^+^CD68^+^ macrophages were linked to pembrolizumab responsiveness, suggesting that TAMs themselves may represent functional targets of ICBs [58]. Along with TAMs, MDSCs can also dampen T-cell responses, leading to ICB resistance [85]. Elevated monocytic-MDSCs (M-MDSCs) and polymorphonuclear-MDSCs (PMN-MDSCs) are frequently detected in pleural effusions and tumor tissues of PM patients [70]. In particular, high intratumoral levels of Tregs, PMN-MDSCs, and M-MDSCs have been independently correlated with poorer outcomes [71,86]. Consequently, strategies to deplete or re-polarize TAMs, such as CSF-1R inhibitors, CD40 agonists, are being explored to potentiate immunotherapy efficacy [87].

The spatial organization of immune cells is another extrinsic determinant that can affect response to immunotherapy. When TILs are sequestered in the stroma and fail to penetrate tumor nests, they cannot kill cancer cells even if activated [88]. This immune exclusion can result from chemokine misalignment or physical barriers, such as dense fibrotic stroma produced by cancer-associated fibroblasts (CAFs) and neoplastic cells undergoing mesothelial-to-mesenchymal transition. Notably, mesotheliomas with desmoplastic histology are particularly resistant to therapy, likely due in part to this T-cell exclusion mechanism [10].

High levels of transforming growth factor β (TGF-β) within the TME—frequently secreted by stromal fibroblasts and M2-polarized macrophages—further reinforce immune exclusion by promoting desmoplasia and limiting T-cell infiltration [89]. In PDAC, for example, TGF-β activation drives CAF differentiation into α-SMA^+^ myofibroblasts, leading to excessive collagen deposition, stromal stiffening and reduced CD8^+^ T-cell access [90]. Consistently, TGF-β blockade in vivo has been shown to remodel CAF subsets, downregulating myofibroblast activity, while expanding IFN-responsive, immunomodulatory fibroblasts, thus facilitating T-cell infiltration and enhancing the efficacy of PD-1 blockade [91]. In mesothelioma, TGF-β1 also drives M2 macrophage polarization, sustaining an immunosuppressive TME [92]. Fittingly, multiple studies have demonstrated that elevated expression of TGF-β1 in the TME of PM correlates with increased levels of mesenchymal markers and M2 macrophage infiltration [93,94]. Importantly, high intra-tumoral levels of TGF-β1 are associated with poor outcomes, irrespective of histology subtype [93,94], thereby suggesting TGF-β1 as a potential therapeutic target in PM.

Altogether, these findings emphasize that the composition, spatial organization, and cytokine milieu of the TME are crucial determinants of ICB responsiveness. Clarifying how these extrinsic factors, in particular stromal architecture and cytokine signaling, affect immune access and activation will be key to developing new strategies that can overcome immune exclusion and enhance the therapeutic efficacy of ICB in PM.

### 5.5. Patient Factors and Systemic Immune State

In addition to TME characteristics, the analysis of circulating immune cell populations has emerged as a minimally invasive and potentially reliable strategy to predict and monitor responses to ICB [95]. Because effective antitumor immunity depends on a competent immune system capable of generating and sustaining cytotoxic T-cell activity, systemic immune fitness and overall inflammatory status critically modulate the efficacy of ICB. This is particularly relevant in PM, where the typical patient population has a median age of about 70 years and often presents with multiple comorbidities, including chronic inflammation, which singularly or collectively can influence immune competence, potentially reducing responsiveness to immunotherapy.

**Figure 5:**
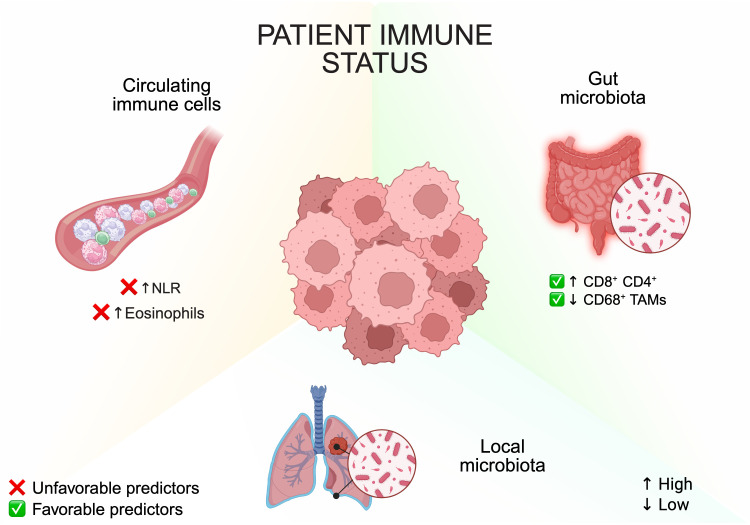
Patient immune status influences response to ICB. The composition of circulating immune cells can serve as an indicator of systemic immune fitness and overall inflammatory status, thereby affecting the efficacy of ICB. Elevated neutrophil-to-lymphocyte ratio (NLR) and higher eosinophil counts are associated with poorer outcomes. Emerging evidence also highlights the importance of microbial ecosystems across multiple anatomical sites in shaping disease progression and treatment response in PM. Specific gut bacterial genera correlate with more favorable TME features, including higher CD8^+^ and CD4^+^ TIL densities, reduced CD68^+^ TAM infiltration, and improved PFS. Local microbial communities (intra-tumoral and pleural) may similarly exert site-specific immunomodulatory effects with prognostic relevance. The figure was created with BioRender (www.BioRender.com, accessed on 9 December 2025).

A typical example of age-related immune decline is represented by progressive T cell dysfunction [96], a chronic low-grade inflammatory state known as inflammaging [97], and a shift in hematopoiesis toward “emergency myelopoiesis” [98]. This latter process, driven by both tumor- and age-derived factors, promotes the aberrant expansion and mobilization of immunosuppressive, pro-tumorigenic myeloid populations, including monocytes, neutrophils, and both M-MDSCs and PMN-MDSCs [98,99]. Because these myeloid subsets inhibit T-cell activation and promote tumor progression, the analysis of circulating myeloid cells and T lymphocytes has emerged as a promising strategy to identify predictive biomarkers of response in ICB-treated patients (Figure 5) [100].

Among systemic markers, the neutrophil-to-lymphocyte ratio (NLR) has gained broad clinical relevance as an indicator of systemic inflammation and immune suppression. Elevated baseline NLR correlates with poorer prognosis in various malignancies, including mesothelioma [101,102]. Based on these observations, the Lung Immune Prognostic Index (LIPI), which combines baseline neutrophil counts with serum lactate dehydrogenase (LDH) levels, has been validated as a reliable prognostic tool for patients undergoing ICB therapy [103,104,105,106], including older individuals [107]. In mesothelioma, patients with favorable LIPI scores experienced longer survival following immunotherapy. However, LIPI did not clearly differentiate the benefit between ICB and chemotherapy treatment groups [16].

Other circulating immune cells also modulate therapeutic outcomes. For instance, elevated baseline eosinophil counts have been associated with worse outcomes in PM patients receiving chemotherapy or immunotherapy [108]. While this observation requires validation in larger, prospective cohorts, growing evidence across multiple cancer types suggests that elevated eosinophil counts, either at baseline or during treatment, may influence ICB efficacy in a context-dependent manner [109].

Another important environmental factor influencing ICB efficacy across multiple cancer types, including melanoma and non–small cell lung cancer (NSCLC), is the microbiome, particularly the gut microbiome [110]. In this regard, evidence from the phase II MiST4 trial (NCT03654833), which evaluated dual inhibition of PD-L1 (atezolizumab) and VEGF (bevacizumab) in 26 patients with relapsed mesothelioma, unveiled a significant association between the specific composition of gut microbial communities and clinical outcomes [32]. Although no significant differences in overall alpha-diversity were observed between responders (Rs) and non-responders (NRs), distinct bacterial genera were enriched in Rs compared to NRs, suggesting that qualitative differences, rather than total microbial diversity, may underlie differential therapeutic responses. Notably, the log odds ratio (LOD) of genus-level enrichment was nearly twofold higher in Rs, and this increase correlated with favorable features of the tumor immune microenvironment, including higher frequencies of CD8^+^ and CD4^+^ TILs, lower densities of CD68^+^ TAMs, and improved PFS [32]. Although validation in larger cohorts is warranted, these early findings support the hypothesis that targeted modulation of the gut microbiota—potentially through dietary or microbial interventions—could reshape the tumor immune microenvironment in mesothelioma and enhance responsiveness to ICB-based therapies.

In addition to the gut, recent studies have explored the role of intra-tumoral and pleural effusion-associated microbiota in modulating clinical outcomes in PM. By analyzing TCGA dataset from 86 PM patients, Pentimalli et al. identified 107 bacterial genera whose presence was significantly associated with patient survival, suggesting that the intra-tumoral microbiota may serve as a novel prognostic indicator [111]. In a separate study, Kwok et al. investigated the microbial composition of malignant, paramalignant, and non-malignant pleural effusions, identifying distinct microbial signatures linked to poor prognosis in thoracic malignancies, including PM and lung cancer [112]. These findings point to the pleural fluid microbiota as a promising, minimally invasive source of diagnostic and prognostic biomarkers.

Although preliminary, these studies collectively underscore the relevance of microbial ecosystems across multiple anatomical compartments, including the gut, tumor tissue, and pleural space, in influencing disease progression and therapeutic responses in PM (Figure 5). In keeping with findings from other cancer types, the gut microbiota appears to modulate systemic immune tone and influence ICB efficacy, whereas local microbial communities (intra-tumoral and pleural) may exert site-specific effects with prognostic significance.

Overall, the clinical efficacy of ICB in PM arises from a complex interplay between tumor-intrinsic and immune-extrinsic factors. This multifaceted scenario supports the development of predictive algorithms that integrate immune cell profiles, neoantigen quality, stromal context, inhibitory checkpoint expression, and microbiome signatures to better stratify patients for immunotherapy. Understanding mechanisms of both primary and acquired resistance will be essential for designing personalized immunotherapeutic combinations.

## 6. Strategies to Enhance ICB Response in PM

Alongside the identification of predictive biomarkers to guide clinical decision-making, numerous strategies are being investigated to bolster the effectiveness of ICB in PM. These approaches aim either to enhance anti-tumor immune response or to disable tumor defenses that cause resistance. Many strategies are tailored to the unique aspects of PM histological subtypes and TME. As a result, the landscape of mesothelioma clinical trials is rapidly evolving.

Here, we focus on key avenues, including notable preclinical studies (Table 3) and trials (Table 4) that are evaluating emerging combination strategies built on the backbone of ICB or exploring their use in new settings, such as earlier-stage disease.

### 6.1. ICB Plus Chemotherapy

The rationale for combining cytotoxic chemotherapy with ICBs extends beyond the potential additive effect on cancer cells and the TME. Certain chemotherapeutic agents can favorably modulate the immune milieu, thereby enhancing responsiveness to immunotherapy [133]. For instance, gemcitabine, 5-fluorouracil, and cyclophosphamide can transiently reduce immunosuppression by depleting MDSCs or Tregs [134]. Others, such as doxorubicin and oxaliplatin, can promote activation of anti-tumor immunity by inducing immunogenic cell death (ICD) [135]. ICD triggers the release of both antigens and adjuvant-like signals (e.g., calreticulin exposure and ATP release), which in turn stimulate tumor-specific adaptive immune responses [135,136]

In PM, chemo-ICB combinations are being actively investigated, particularly in epithelioid tumors, which are more responsive to platinum–pemetrexed chemotherapy [28]. In this setting, chemotherapy can debulk tumors and expose antigens, while ICB sustains long-term immune control. Importantly, this approach may help counter the early disease progression observed with ICB monotherapy in some epithelioid cases, as judged by the shorter PFS observed in the ICB arm of CheckMate 743, likely due to a lack of immediate cytoreduction.

Several single-arm phase II studies have reported encouraging results with platinum–pemetrexed plus NIVO (anti-PD1) (JME-001 trial) [118] or durvalumab (anti-PD-L1) (PrE0505 and DREAM trial) [25,26] in the first-line setting. Despite the small sample size (n = 18), the JME-001 trial (NIVO + chemotherapy; N = 18) showed that adding NIVO to chemotherapy improved outcomes compared with historical chemotherapy-alone data [118]. Similarly, in the PrE0505 study, the addition of durvalumab to chemotherapy yielded an ORR of ~56% (predominantly in epithelioid tumors), median OS of ~20.4 months vs. 12.1 months expected with chemotherapy alone, and a 1-year OS rate of 70% [26]. The DREAM trial (durvalumab + chemotherapy) also showed improved outcomes compared to historical controls, with a 6-month PFS rate of 57%, ORR of 48% (vs. ~20–25% historically), and median PFS of 6.9 vs. 5.7 months [25]. More recently, the phase III IND-227 trial demonstrated superior efficacy of pembrolizumab plus platinum-based chemotherapy compared with chemotherapy alone in 440 patients: the combination achieved a 62% ORR and median OS of 17.3 months vs. 16.1 months [56]. These results support further phase III evaluation of chemo-immotherapy, such as the ongoing DREAM3R/PrE0506 trial (NCT04334759), which directly compares chemo–durvalumab with chemotherapy alone in first-line PM [119].

Anti-angiogenic agents also represent a promising avenue to optimize the TME, with clinical trials such as MAPS, LUME-Meso, and RAMES showing improved outcomes when these agents are combined with chemotherapy [137,138,139]. By promoting vascular normalization and immune cell infiltration, these agents may also potentiate ICB efficacy. Indeed, combined blockade of PD-L1 and VEGFR2 led to promising results in preclinical mesothelioma models [115]. In clinical settings, the BEAT-meso trial (NCT03762018) evaluated atezolizumab in combination with bevacizumab, carboplatin, and pemetrexed in advanced PM. While the primary endpoint was not met, prespecified analyses showed improved OS and PFS for the quadruplet regimen in non-epithelioid subtypes [120].

Collectively, early-phase trials have brought encouraging results, but definitive evidence and optimal integration of chemo–immunotherapy, alone or in combination with anti-angiogenic strategies, are still under investigation. A likely therapeutic paradigm may emerge in which chemo-ICB combinations are favored for epithelioid tumors, particularly in patients suitable for intensive treatment, whereas dual ICB or chemo–immunotherapy combined with anti-angiogenic drugs could offer greater benefit in non-epithelioid subtypes.

### 6.2. Dual and Triple Checkpoint Blockade

Following the clinical success of combined PD-1 and CTLA-4 inhibition, additional immune checkpoints are being explored as therapeutic targets. Among these, LAG-3 has attracted particular attention after the approval of NIVO plus relatlimab (anti–LAG-3) for melanoma and the launch of multiple trials in other solid malignancies [113]. In a preclinical mesothelioma model, dual blockade with anti-PD-L1 and anti-LAG-3 delayed tumor growth and improved survival compared with controls, although the combination did not outperform anti-PD-L1 monotherapy [114]. Consistent with these findings, analyses of human mesothelioma samples often show co-expression of LAG-3 and PD-1 on exhausted TILs. Moreover, elevated LAG-3 mRNA expression has been described as an independent prognostic biomarker in PM [140] and, as described in Section 5.2, when incorporated into a four-gene inflammatory signature as a predictor of better outcomes in patients treated with ICB [13]. These findings support continued evaluation of dual blockade regimens, such as nivolumab plus relatlimab, in mesothelioma.

Additional inhibitory checkpoints under investigation include TIM-3 and TIGIT, both of which are being evaluated in clinical trials for advanced solid tumors, though mesothelioma-specific data are not yet available [141]. Nevertheless, growing evidence suggests that dual blockade of PD-1/PD-L1 and TIGIT holds promise as an effective immunotherapy for PM [114,142]. In an epithelioid mesothelioma model, anti-PD-1 plus anti-TIGIT induced stronger and more durable anti-tumor responses than either chemotherapy or dual anti-PD-1 plus CTLA-4 therapy [114]. Clinical evidence also supports this approach, as shown in the phase I AdvanTIG-105 study, in which two patients with epithelioid PM achieved partial responses after receiving tislelizumab (anti-PD-1) and ociperlimab (anti-TIGIT) [121]. These results highlight the need for dedicated clinical trials to evaluate anti-PD-1/PD-L1 plus anti-TIGIT therapy, particularly in epithelioid mesothelioma.

VISTA is another emerging checkpoint of interest in cancer immunotherapy [143], being highly expressed in epithelioid subtypes. Considering that several anti-VISTA antibodies (e.g., CI-8993, HMBD-002, and KVA12123) are currently undergoing early-phase trials for solid tumors [144], both monotherapy and combination strategies with anti-PD-1/PD-L1 warrant investigation in epithelioid mesothelioma.

Finally, triple checkpoint blockade, such as the combination of PD-1 and CTLA-4 inhibitors with a third target like LAG-3 or TIGIT, represents a highly aggressive but potentially potent strategy for refractory or immunologically “cold” tumors with limited T-cell infiltration. However, toxicity remains a major concern, as PD-1 plus CTLA-4 alone already doubles the rate of immune-related adverse events, underscoring the importance of careful patient selection and optimized dosing strategies.

### 6.3. Targeting Immunosuppressive Cells: Macrophages and Tregs

TAMs are the most abundant immune population in the mesothelioma microenvironment and act as key orchestrators of immunosuppression. Consequently, strategies aimed at either depleting TAMs or reprogramming them from an M2- to an M1-like phenotype are sought-after means of enhancing immunotherapy efficacy.

In this scenario, combined PD-1 and CSF-1R blockade is being tested across multiple cancer types [145] and may hold value for PM as well. Preclinical studies show that CSF-1R inhibition, when paired with PD-1 blockade in both human multicellular spheroid models of PM [146] and murine models of sarcomatoid PM, enhances T-cell activity by reducing suppressive myeloid cells [84]. More recently, a novel soluble human CSF-1R protein capable of neutralizing both CSF-1 and IL-34 demonstrated superior activity in vitro, effectively blocking monocyte survival and inhibiting TAM differentiation [147]. These data provide a solid preclinical rationale for advancing CSF-1R–based combination therapies into clinical evaluation for mesothelioma.

CD40 agonists, such as selicrelumab and sotigalimab, represent another immunotherapeutic strategy under active investigation as monotherapy or in combination with ICB [148]. By engaging both adaptive (B cells) and innate (macrophages, DCs) immune cells, CD40 agonists can reprogram the TME from an immunosuppressive to an immunostimulatory state, thus providing a strong rationale for their integration with ICB [149]. Although clinical trials in mesothelioma are still limited, preclinical studies in murine models have demonstrated efficacy of CD40 agonists as monotherapy [150] not just in adjuvant settings post-surgery [151] but also in combination with other immunostimulatory agents, such as TLR ligands and IL-2 [152,153,154,155]. Translational evidence from early-phase trials further supports their potential. In a phase Ib trial, the CD40 agonist CP-870,893 combined with standard chemotherapy (cisplatin and pemetrexed) led to favorable immune activation in PM [156]. To further enhance TME-dependent CD40 activation while minimizing systemic toxicity, a first-in-class mesothelin–CD40 bispecific antibody was recently engineered and tested in patients with tumors expressing high levels of mesothelin, such as PM and ovarian cancer. Results from the phase I study demonstrated safety, tolerability, and preliminary anti-tumor activity, supporting further evaluation in larger patient cohorts [157].

Tregs also play a pivotal role in maintaining the immunosuppressive TME in PM. Depletion strategies using low-dose cyclophosphamide have been mostly evaluated in combination with DC vaccines [158]. In a preclinical model of mesothelioma, as well as in a small-sized phase I trial, the combination of low-dose cyclophosphamide administered after vaccination with autologous tumor lysate-pulsed DCs (NCT 01241682) proved feasible, safe, and showed encouraging signs of clinical activity [159]. Notably, a single low-dose (metronomic) of cyclophosphamide given before vaccination significantly reduced circulating Tregs and promoted expansion of effector and effector memory T cells [160]. Despite the small sample size, these promising results support continued investigation of cyclophosphamide-induced Treg depletion as a strategy to mitigate immunosuppression, thereby enhancing the efficacy of cancer immunotherapies, such as cancer vaccines, but also ICB.

### 6.4. Anti-Angiogenic and Stroma-Targeting Therapy

VEGF is highly expressed in pleural mesothelioma and plays a dual role in tumor biology by promoting angiogenesis and exerting potent immunosuppressive effects. VEGF inhibits DC maturation while stimulating the proliferation of Tregs and MDSCs, thereby dampening antitumor immune responses. These properties provide a strong rationale for combining VEGF blockade with ICB. In addition to the Beat-Meso trial, which evaluated the triple combination of bevacizumab, atezolizumab, and chemotherapy [120], other studies suggest that VEGF inhibition combined with ICB alone may also yield therapeutic benefit, as demonstrated in other solid tumors such as renal cell and hepatocellular carcinomas [161].

In mesothelioma, two single-arm phase II trials (i.e., PEMMELA and HCRN-LUN15-299) evaluated ICB plus anti-angiogenic strategies in the second-line setting. The combination of lenvatinib, a multikinase anti-angiogenic inhibitor, with pembrolizumab (anti-PD-1) showed significant antitumor activity, although associated with considerable toxicity [123]. Similarly, ramucirumab (anti-VEGFR2) plus NIVO (anti-PD-1) resulted in favorable clinical outcomes in non-epithelioid mesothelioma compared with historical data for NIVO monotherapy, with an acceptable safety profile and no grade ≥ 4 toxicities observed [122]. Collectively, these studies indicate that targeting VEGF may help overcome immune resistance in mesothelioma and call for further evaluation in combination with ICB in larger randomized trials.

In addition to abnormal tumor vasculature, the extensive stromal compartment of PM poses a major barrier to immune cell infiltration, thus hindering the efficacy of ICB. Even though no therapies specifically targeting CAFs have yet been developed for mesothelioma, several promising strategies from other desmoplastic solid tumors such as PDAC, may be applicable [162]. Agents such as hyaluronidase, which depletes hyaluronan, and focal adhesion kinase (FAK) inhibitors, which also modulate CAF activity, have been shown to disrupt stromal barriers, enhance immune infiltration, and improve ICB efficacy in PDAC models [163,164,165,166]. A phase I/IIA trial has recently tested the combination of the FAK inhibitor defactinib with pembrolizumab in advanced solid tumors, including mesothelioma, though results are not yet available [125].

Another stroma-targeting approach involves inhibiting TGF-β, which plays a central role in both fibrotic remodeling and immune suppression. TGF-β neutralization may reduce stromal barriers, facilitate T-cell infiltration, restore antitumor immune activity, thereby overcoming resistance to ICB [167]. Although a phase II study of the anti–TGF-β antibody GC1008 in mesothelioma was prematurely terminated despite preliminary evidence of activity [168], dual blockade of TGF-β and PD-1/PD-L1 is under evaluation in various solid tumors. However, mesothelioma-specific data are not yet available [167].

Taken together, these findings highlight stromal targeting strategies, including CAF modulation, FAK inhibition, or TGF-β neutralization, as a compelling yet underexplored strategy to overcome immune resistance.

### 6.5. Epigenetic Modulators

Epigenetic dysregulation is a hallmark of cancer, including PM [169]. Besides driving aberrant transcriptional programs that promote tumor initiation and progression, epigenetic remodeling can also diminish tumor cell immunogenicity [170]. Consequently, pharmacologic modulation of the epigenome has gained attention as a strategy to enhance tumor immune recognition and restore sensitivity to ICB [171].

Among epigenetic regulators, enhancer of zeste homolog 2 (EZH2), the catalytic subunit of the polycomb repressive complex 2 (PRC2), acts as an oncogenic driver in several malignancies, including PM [172,173,174]. Preclinical studies have shown that EZH2 inhibition suppresses tumor growth, particularly in PM harboring *BAP1* loss. However, the encouraging preclinical efficacy has been only partially confirmed in early clinical trials with tazemetostat, an EZH2 inhibitor approved for advanced epithelioid sarcoma and relapsed or refractory follicular lymphoma [175,176]. These findings underscore the need to better define the molecular determinants underlying PM sensitivity to tazemetostat, as well as the immunomodulatory effects of EZH2 inhibition.

EZH2 inhibitors exert multifaceted effects on tumor immunity. In several cancers, including melanoma, head and neck, and prostate tumors, EZH2 inhibition is associated with increased tumor immunogenicity and enhanced responsiveness to CTLA-4 or PD-1/PD-L1 blockade [177,178,179]. Mechanistically, EZH2 inhibition enhances antigen presentation, activates IFN signaling, and reverses adaptive resistance to immunotherapy [180]. Furthermore, EZH2 inhibitors can induce complex immune remodeling within the TME. On one hand, they can increase the recruitment and activation of cytotoxic immune cells by upregulating chemokines (e.g., CXCL9, CXCL10) and NKG2D ligands, as shown in bladder cancer [181], PDAC [182], colon cancer [183], hepatocellular carcinoma [184], and glioblastoma [185]. On the other hand, EZH2 inhibition may also promote expansion and intratumoral accumulation of immunosuppressive myeloid populations. Preclinical studies in lung and colon cancer show that EZH2 inhibitors can drive the expansion of MDSCs [186], while studies in mesothelioma [187] and breast cancer [188] have reported increased recruitment and polarization of pro-tumorigenic M2 macrophages. Therefore, rational combination approaches that include EZH2 inhibitors alongside strategies to deplete or reprogram immunosuppressive myeloid cells may enhance antitumor immunity and improve ICB efficacy [189].

Other classes of epigenetic drugs, including DNA methyltransferase inhibitors (DNMTis, such as decitabine) and histone deacetylase (HDAC) inhibitors, have also demonstrated potential to convert immunologically “cold” tumors into “hot” ones. These agents enhance the expression of tumor-associated antigens (e.g., NY-ESO-1) and endogenous retroviral elements, leading to activation of type I IFN responses [170]. In a recent phase I dose-escalation study (NCT02998567), the combination of the DNMT inhibitor guadecitabine with pembrolizumab was evaluated in patients with advanced solid tumors, including PM. The results indicated that the regimen was well tolerated and that DNA hypomethylating therapy could potentially overcome resistance to prior ICB treatment [124].

In summary, these findings highlight the therapeutic promise of epigenetic modulators in mesothelioma, both as single agents and in combination with immunotherapy. Future studies should focus on defining the context-dependent immunological consequences of epigenetic reprogramming to guide rational combinations that maximize antitumor efficacy while minimizing adverse immune effects.

### 6.6. Tumor Vaccines and Cell Therapies

Combining cancer vaccines with ICB represents an emerging strategy to overcome the limitations of each approach and to achieve synergistic antitumor activity. Cancer vaccines can prime the immune system against mesothelioma-specific antigens, thereby increasing the pool of tumor-infiltrating T cells. However, despite the limited clinical efficacy of vaccine-induced T cell responses when used alone, they can significantly enhance the effectiveness of subsequent ICB by converting immunologically inactive tumors into more responsive ones.

Several phase I/II clinical trials have investigated DC-based vaccines loaded with either autologous tumor lysates or allogeneic mesothelioma cell line lysates, showing encouraging results in terms of disease control and prolonged survival [190]. However, in the subsequent phase II/III DENIM trial, the allogeneic DC vaccine MesoPher combined with best supportive care failed to demonstrate clinical benefit compared with standard care alone [191]. Despite this result, both preclinical and early clinical data indicate that DC vaccination can act synergistically with PD-1/PD-L1 inhibitors [126]. In good agreement with these findings, the ongoing multicenter, single-arm phase I/II Immuno-MESODEC trial (NCT05765084) is assessing the integration of PD-L1 inhibition (atezolizumab) and WT1/DC vaccination with standard platinum–pemetrexed chemotherapy in the first-line treatment of epithelioid PM [126]. Similarly, the open-label NIPU trial evaluated the telomerase peptide vaccine UV1 in combination with IPI + NIVO in previously treated PM patients. Although the study did not meet its primary endpoint of PFS, predefined analyses of response and survival outcomes favored the triplet combination, supporting further exploration in the first-line setting [127].

Adoptive cell therapies represent another innovative immunotherapeutic approach with potential synergy with ICB. A phase I pilot study of autologous TIL therapy in mesothelioma (NCT02414945) was initiated nearly a decade ago, although efficacy results are still pending. Regardless, the clinical application of TIL therapy in mesothelioma faces substantial logistical challenges, including limited cell yields, complex expansion protocols, and reliance on IL-2 support, compounded by the low surgical eligibility rate of PM patients.

Conversely, chimeric antigen receptor (CAR) T-cell therapy is advancing rapidly in PM [192]. Several CAR-T constructs targeting mesothelin—a surface glycoprotein highly expressed in epithelioid mesothelioma but largely absent from normal mesothelial cells—have shown encouraging early clinical signals [193,194]. In a phase I trial, intrapleural administration of anti-mesothelin CAR-T cells achieved disease stabilization and appeared to potentiate antitumor immunity when followed by PD-1 blockade [128], suggesting that PD-1 inhibition can prevent CAR-T cell exhaustion and amplify immune responses initiated by CAR-T-mediated tumor cell lysis.

Ongoing trials (NCT04577326, NCT03054298) are currently assessing mesothelin-targeted CAR-T cell therapy in PM and other mesothelin-expressing malignancies. In addition, CAR-T cells targeting podoplanin, a transmembrane glycoprotein highly expressed in sarcomatoid mesothelioma, have shown antitumor activity in preclinical glioblastoma models [195] and may represent a valuable therapeutic option for sarcomatoid mesothelioma, which typically expresses high levels of this surface protein, but low levels of mesothelin.

Altogether, tumor vaccines and cell-based therapies, particularly when rationally integrated with ICB, represent a rapidly evolving frontier in mesothelioma treatment. While still in early clinical development, these strategies hold potential to achieve durable disease control and could become integral components of future multimodal therapeutic regimens.

### 6.7. Oncolytic Viruses and Local Therapies

The use of viruses that selectively infect and lyse tumor cells while stimulating antitumor immune responses represents an attractive strategy for “immune desert” tumors like PM. The anatomic confinement of PM and its often localized growth pattern make it particularly amenable to direct intratumoral or intrapleural viral administration [196]. Preclinical studies have provided compelling evidence that oncolytic viruses can effectively induce immunogenic tumor cell death and enhance antitumor immunity in mesothelioma [197,198]. Based on these findings, a randomized phase II clinical trial recently evaluated intrapleural administration of ONCOS-102, an oncolytic adenovirus encoding GM-CSF, in combination with standard chemotherapy vs. chemotherapy alone. Although the study did not meet its primary efficacy endpoints, ONCOS-102 was well tolerated and elicited enhanced T-cell infiltration within the TME, correlating with improved survival outcomes [199], providing a strong rationale for combining oncolytic virotherapy with ICB. Supporting this approach, preclinical models have demonstrated potent synergistic antitumor effects from the combination of an engineered oncolytic adenoviral vector (AdV5/3-D24-ICOSL-CD40L) and an anti-PD-1 monoclonal antibody [116].

Insights from other solid tumors further reinforce the therapeutic potential of such combinations, particularly in treatment-resistant settings. In advanced melanoma, for example, the addition of talimogene laherparepvec (T-VEC) to pembrolizumab did not improve clinical outcomes in treatment-naïve patients [200]. However, in PD-1-refractory melanoma, the same combination demonstrated antitumor activity and tolerability, suggesting a role for oncolytic viruses in reversing immunotherapy resistance.

Radiotherapy may also act as an immunologic adjuvant that potentiates ICB. In a preclinical murine model of mesothelioma, low-dose, low-fraction radiotherapy promoted vascular normalization and immune cell infiltration, thus enhancing ICB efficacy [117,201,202]. In addition to its local effects, radiotherapy may also trigger systemic antitumor immune responses, known as abscopal effects. Two independent case reports have documented abscopal responses in patients with PM treated with palliative radiotherapy combined with pembrolizumab, resulting in improved clinical outcomes [129,130]. Further supporting the potential synergy between radiotherapy and ICB, analysis of peripheral blood samples from 35 patients with PM undergoing radical hemithorax radiotherapy (RHRT) revealed marked immunomodulatory effects, including increased frequencies of PD-1^+^ and proliferating T cells, expansion of T-cell clones, and elevated levels of soluble PD-L1 [203]. Given its demonstrated histotype-independent clinical benefits, RHRT may serve as a valuable component of multimodal strategies, potentially sensitizing epithelioid PM to subsequent maintenance immunotherapy.

Following the success of both neo-adjuvant and adjuvant immunotherapy in NSCLC [204,205,206,207], several trials are currently investigating the integration of ICB with surgery in PM patients [208,209]. In the neoadjuvant setting, durvalumab alone or in combination with tremelimumab increased intratumoral CD8^+^ T-cell infiltration in PM tumors, though the CD8^+^/Treg ratio remained unchanged, and survival was longer among patients who subsequently received adjuvant chemotherapy [131]. A phase II trial evaluating neoadjuvant NIVO or IPI + NIVO in resectable PM confirmed feasibility and suggested that circulating tumor DNA (ctDNA) analysis may help assess minimal residual disease [132]. Additional ongoing trials (NCT03918252, NCT06155279, NCT05647265, NCT05932199, NCT04996017) are exploring various perioperative treatment combinations and endpoints, as comprehensively summarized in recent reviews [208,209]. Similarly, the current state of hyperthermic intrathoracic chemoperfusion (HITHOC) as an adjunct to cytoreductive surgery, as well as additional clinical trials evaluating this multimodal approach in combination with post-surgical immunotherapy, have been thoroughly reviewed by Kong S. L. and colleagues [210].

Collectively, these studies underscore the growing interest in integrating immunotherapy into multimodal treatment strategies for PM. Although consistent survival gains have not yet been confirmed, the feasibility and early biological signals of neoadjuvant and adjuvant ICB, as well as its potential combination with locoregional approaches—including oncolytic virotherapy, radiotherapy, cytoreductive surgery, and HITHOC—support continued clinical investigation. Current and future trials will ultimately determine how perioperative immunotherapy can be optimally incorporated into standard-of-care regimens to achieve durable disease control and meaningful clinical benefit for patients with PM.

## 7. Conclusions and Future Directions

The introduction of ICB has opened new therapeutic avenues for PM, demonstrating that even “cold,” poorly immunogenic tumors can benefit from immunotherapy. Despite this breakthrough, clinical outcomes remain suboptimal, underscoring the need for a deeper understanding of the molecular and immunological mechanisms dictating response and resistance to ICB. The pronounced histological, molecular, and immune heterogeneity of PM indicates that a one-size-fits-all strategy is unlikely to deliver optimal results. This review highlights histological subtype as a major determinant of immunotherapy response, suggesting that histology-tailored approaches combined with an in-depth understanding of subtype-specific molecular and immune features represent key future directions. Notably, while non-epithelioid tumors—once uniformly fatal within a year—now demonstrate durable benefit with dual checkpoint blockade, epithelioid tumors derive only modest advantage, warranting multimodal strategies combining ICB with conventional treatments (i.e., chemotherapy, radiotherapy, and surgery), novel checkpoints, or other immunomodulatory agents to enhance anti-tumor immunity.

Emerging evidence from biomarker studies and resistance analyses further emphasizes the need for integrated predictive models. No single biomarker (e.g., PD-L1 expression or tumor mutational burden) adequately predicts response. Instead, composite and spatial biomarkers, such as immune gene expression signatures, T-cell receptor clonality, and spatial mapping of immune infiltration, offer greater predictive value. For example, immune-excluded tumors might benefit from stroma-targeting combinations, whereas inflamed, PD-L1-high tumors could be triaged to dual ICB alone. Liquid biopsy-based markers, including circulating immune cell subsets and ctDNA, may complement tissue-based assays for real-time response monitoring. The immunomodulatory effects of the gut and local microbiota (intratumoral or intrapleural) represent another promising area, both as predictors of ICB response and as actionable therapeutic targets. Moreover, unresolved questions—such as the role of BAP1 status, biphasic differentiation, and hyperprogression—remain priorities for translational research. While BAP1 mutations may enhance chemosensitivity, their impact on immunotherapy remains unclear. Multiomic approaches will be essential to elucidate how tumor-intrinsic genomic and epigenetic alterations shape the immune landscape and influence ICB response. Since the benefit of ICB in biphasic tumors may depend on the extent of sarcomatoid differentiation, integrating refined pathological and transcriptomic analyses could guide treatment decisions. Finally, better characterization of hyperprogression—a rare but serious phenomenon of accelerated tumor growth under ICB—is essential to identify at-risk patients before therapy initiation.

From a therapeutic standpoint, future research will likely advance along several parallel tracks: (i) optimizing combination strategies; (ii) refining biomarkers to guide treatment selection; (iii) identifying novel therapeutic targets; and (iv) defining maintenance and surveillance regimens. Rational combinations that synergize distinct mechanisms of immune activation will continue to dominate, though balancing efficacy with toxicity and cost remains critical. The interplay between local and systemic therapies, such as surgery, radiotherapy, and intrapleural approaches, with immunotherapy represents a particularly promising frontier. Integrating precise surgical resection, strategic radiation or regional therapy to eliminate disease reservoirs or promote immunogenic cell death, and systemic therapies to eradicate microscopic metastases could bring about substantial clinical gains.

In the near future, multiomic tumor profiling will enable more personalized immunotherapy. Patients with inflamed, immunogenic tumors may benefit from PD-1 blockade alone and avoid added toxicity, whereas those with dominant immune-evasion pathways may require tailored combinations that restore immune recognition and enhance tumor clearance. The discovery of novel targets and therapeutic formats, such as bispecific T-cell engagers, antibody–drug conjugates, and engineered cytokines, is expected to expand available treatment options when integrated with ICB. For patients achieving durable responses, determining the optimal treatment duration and the role of maintenance strategies, including vaccines or cytokine-based agents, remains an important research focus.

In conclusion, considerable progress has been achieved in advancing the immunotherapy of mesothelioma, yet significant challenges persist. Deeper insight into the determinants of ICB response—spanning histologic patterns, genetic mutations, and immune contexture—is already driving the development of innovative, biomarker-guided trials and rational combination regimens. With continued multidisciplinary collaboration, these efforts may ultimately transform mesothelioma from an intractable malignancy into a disease amenable to durable immune control and, potentially, long-term remission.

## 8. Conclusions

• ICB has expanded treatment options for PM, demonstrating activity even in poorly immunogenic tumors. However, overall responses remain modest, largely due to the strong heterogeneity of PM at the histologic, molecular, and immune levels.
• Histology clearly influences therapeutic benefit. Non-epithelioid tumors derive the greatest advantage from dual ICB, whereas epithelioid tumors show only limited improvement. For these patients, multimodal strategies integrating ICB with chemotherapy, radiotherapy, surgery, or new immunomodulatory agents may be required.
• Predicting ICB response will depend on integrated rather than single biomarkers. Composite and spatial immune signatures, liquid biopsy markers, and microbiome-related features appear more informative than PD-L1 or TMB alone, offering more refined tools for patient stratification.
• Several biological questions remain unresolved, including the immunologic impact of BAP1 mutations, the role of sarcomatoid differentiation in biphasic tumors, and the mechanisms underlying hyperprogression. Multiomic approaches will be essential to clarify how tumor-intrinsic alterations shape immune responses.
• Future progress will depend on optimizing combination therapies, refining biomarker-guided treatment selection, and identifying new immunotherapy targets. Defining appropriate maintenance strategies for long-term responders will also be important as more patients achieve durable benefit.
• Overall, advances in tumor profiling and rational treatment integration may gradually shift mesothelioma toward a disease more amenable to durable immune control and, potentially, long-term remission.

## Figures and Tables

**Table 2:** Genomic and TME differences across histologic subtype.

Molecular/Cellular Features	Epithelioid	Sarcomatoid	Biphasic	Clinical Relevance	Ref
BAP1 loss	≈60–65%	≈25–45%	≈50%	IFN signaling; predictive unclear	[29,34,35]
CDKN2A del	≈40–50%	≈65–75%	≈55–60%	Worse OS; PD-1 resistance signal	[11,34,36]
NF2 mutation	≈35%	≈40%	≈38%	YAP activation; PD-L1 high	[34,37,38]
TP53 mutation	<10%	≈15–20%	≈12%	Poor prognosis	[30,33]
PD - L1 ≥ 1%	10–20%	40–60%	25–35%	Predictive value mixed	[39,40]
VISTA high	Common	Rare	Intermediate	Alternate checkpoint	[31,33]
T-cell pattern	Stromal CD4 > CD8	Intratumoral CD8 high	Heterogeneous	Affects ICI	[8,31,41,42]
M2 macrophages	Moderate	High	High	Correlate with resistance	[8,31]
Stroma/Fibrosis	Dense collagen	Fibrotic, hypoxic	Variable	Barrier	[27]
Median OS (chemo)	≈18–20 mo	≈8–10 mo	≈12–15 mo	Baseline	[8,23,28,29]

**Table 3:** Preclinical evidence supporting combination strategies that enhance ICB efficacy in PM.

Strategy	Regimen/Agents	Rationale	Histotype	Ref.
Dual ICB	Anti-PD-L1 + anti-LAG3	Dual checkpoint blockade	Sarcomatoid	[113]
Dual ICB	anti-PD-1 + anti-TIGIT	Dual checkpoint blockade	Epithelioid	[114]
ICB + Anti-VEGF	Anti-VEGFR2 (clone DC101) + anti-PD-L1 (clone 10F.9G2)	Vessel normalization	Sarcomatoid	[115]
ICB + CSF-1R inhibitor	Pembrolizumab + CSF-1R inhibitor	TAM reprogramming	Sarcomatoid	[84]
Oncolytic virus + ICB	AdV5/3-D24-ICOSL-CD40L + anti-PD-1	Combining oncolytic virotherapy with ICB	Epithelioid	[116]
ICB + radio-therapy	Low-dose, low-fraction radiotherapy	promote immune cell infiltration	Sarcomatoid	[117]

**Table 4:** Clinical trials investigating combination strategies to enhance ICB efficacy in PM.

Strategy	Regimen/Agents	Rationale	Histotype	Identifier	Status	Ref.
Chemo + anti-PD-(L)1	Platinum/peme trexed + nivolumab	Immunogenic cell death; tumor debulking; sustained immune control	Epithelioid	JME-001	Phase II	[118]
Chemo + anti-PD-L1	Platinum/peme-trexed + durvalumab	Immunogenic cell death; debulking; improved ORR/OS	Epithelioid	PrE0505	Phase II	[26]
Chemo + anti-PD-L1	Platinum/peme-trexed + durvalumab	Immunogenic cell death; antigen release; long-term control	Epithelioid	DREAM	Phase II	[25]
Chemo + anti-PD-1	Pembrolizumab + platinum-based chemotherapy	Additive cytotoxic + immune synergy	Epithelioid	(IND-227)	Phase III	[56]
Chemo + anti-PD-L1	Platinum/peme-trexed + durvalumab	Tumor debulking + ICB maintenance	Epithelioid	DREAM3R/PrE0506; NCT04334759	Phase III	[119]
Chemo + anti-PD-L1 + antiVEGF	Atezolizumab + bevacizumab + carboplatin + pemetrexed	Vascular normalization; improved immune infiltration	Non-epithelioid	BEAT-meso; NCT03762018	Phase III	[120]
Anti-PD-1 + anti-TIGIT	Tislelizumab + ociperlimab	Dual checkpoint blockade; overcome PD-1 resistance	Epithelioid	AdvanTIG-105	Phase I	[121]
Anti-PD-1 + anti-VEGFR2	Nivolumab + ramucirumab	Overcome immune resistance via VEGF inhibition	Non-epithelioid	HCRN-LUN15-299	Phase II	[122]
Anti-PD-1 + anti- angiogenic TKI	Pembrolizumab + lenvatinib	Immune modulation via VEGF blockade	All histotypes	PEMMELA	Phase II	[123]
DNMT inhibitor + anti–PD-1	Guadecitabine + pembrolizumab	Epigenetic reprogramming; restore immunogenicity	PM and advanced solid tumors	NCT02998567	Phase I	[124]
FAK inhibitor + ICB	Defactinib + pembrolizumab	Disrupt tumor stroma; enhance immune infiltration	PM and advanced solid tumors	NCT02758587	Phase I/IIA (ongoing)	[125]
DC vaccine + ICB + chemotherapy	WT1/DC vaccine + atezolizumab + chemotherapy	Increase TILs	Epithelioid	Immuno-MESODEC; NCT05765084	Phase I/II (ongoing)	[126]
Peptide vaccine + dual ICB	UV1 (telomerase vaccine) + ipilimumab + nivolumab	Enhance T cell priming	Previously treated PM	NIPU trial	Phase II	[127]
CAR-T + ICB	Intrapleural anti-mesothelin CAR-T followed by PD-1 blockade	Prevent CAR-T exhaustion; amplify immune response	Epitheloid	NCT02414269	Phase I	[128]
ICB + Radio therapy	Radiotherapy + Pembrolizumab	Abscopal effects	Not specified	case report		[129,130]
Neo adjuvant ICB	Durvalumab ± tremelimumab	Increase intratumoral CD8^+^ T cells pre-surgery	Resectable PM	NCT02592551	Phase II	[131]
Neo adjuvant ICB	Nivolumab or ipilimumab + nivolumab	Increase anti-tumor immunity pre-surgery	Resectable PM	NCT03918252	Phase II	[132]

## References

[1] Chen Z., Cai Y., Ou T., Zhou H., Li H., Wang Z., Cai K. (2024). Global Burden of Mesothelioma Attributable to Occupational Asbestos Exposure in 204 Countries and Territories: 1990–2019. J. Cancer Res. Clin. Oncol..

[2] Lopci E. (2025). Current Status of Staging and Restaging Malignant Pleural Mesothelioma. Semin. Nucl. Med..

[3] Brcic L., Kern I. (2020). Clinical Significance of Histologic Subtyping of Malignant Pleural Mesothelioma. Transl. Lung Cancer Res..

[4] Baas P., Scherpereel A., Nowak A.K., Fujimoto N., Peters S., Tsao A.S., Mansfield A.S., Popat S., Jahan T., Antonia S. (2021). First-Line Nivolumab plus Ipilimumab in Unresectable Malignant Pleural Mesothelioma (CheckMate 743): A Multicentre, Randomised, Open-Label, Phase 3 Trial. Lancet Lond. Engl..

[5] Iwai Y., Hamanishi J., Chamoto K., Honjo T. (2017). Cancer Immunotherapies Targeting the PD-1 Signaling Pathway. J. Biomed. Sci..

[6] Krummel M.F., Allison J.P. (1995). CD28 and CTLA-4 Have Opposing Effects on the Response of T Cells to Stimulation. J. Exp. Med..

[7] Ishida Y. (2020). PD-1: Its Discovery, Involvement in Cancer Immunotherapy, and Beyond. Cells.

[8] Perrino M., De Vincenzo F., Cordua N., Borea F., Aliprandi M., Santoro A., Zucali P.A. (2023). Immunotherapy with Immune Checkpoint Inhibitors and Predictive Biomarkers in Malignant Mesothelioma: Work Still in Progress. Front. Immunol..

[9] Scherpereel A., Antonia S., Bautista Y., Grossi F., Kowalski D., Zalcman G., Nowak A.K., Fujimoto N., Peters S., Tsao A.S. (2022). First-Line Nivolumab plus Ipilimumab versus Chemotherapy for the Treatment of Unresectable Malignant Pleural Mesothelioma: Patient-Reported Outcomes in CheckMate 743. Lung Cancer Amst. Neth..

[10] Chu G.J., van Zandwijk N., Rasko J.E.J. (2019). The Immune Microenvironment in Mesothelioma: Mechanisms of Resistance to Immunotherapy. Front. Oncol..

[11] Yang H., Xu D., Schmid R.A., Peng R.-W. (2020). Biomarker-Guided Targeted and Immunotherapies in Malignant Pleural Mesothelioma. Ther. Adv. Med. Oncol..

[12] Alley E.W., Lopez J., Santoro A., Morosky A., Saraf S., Piperdi B., Van Brummelen E. (2017). Clinical Safety and Activity of Pembrolizumab in Patients with Malignant Pleural Mesothelioma (KEYNOTE-028): Preliminary Results from a Non-Randomised, Open-Label, Phase 1b Trial. Lancet Oncol..

[13] Yap T.A., Nakagawa K., Fujimoto N., Kuribayashi K., Guren T.K., Calabrò L., Shapira-Frommer R., Gao B., Kao S., Matos I. (2021). Efficacy and Safety of Pembrolizumab in Patients with Advanced Mesothelioma in the Open-Label, Single-Arm, Phase 2 KEYNOTE-158 Study. Lancet Respir. Med..

[14] Okada M., Kijima T., Aoe K., Kato T., Fujimoto N., Nakagawa K., Takeda Y., Hida T., Kanai K., Imamura F. (2019). Clinical Efficacy and Safety of Nivolumab: Results of a Multicenter, Open-Label, Single-Arm, Japanese Phase II Study in Malignant Pleural Mesothelioma (MERIT). Clin. Cancer Res..

[15] Fennell D.A., Ewings S., Ottensmeier C., Califano R., Hanna G.G., Hill K., Danson S., Steele N., Nye M., Johnson L. (2021). Nivolumab versus Placebo in Patients with Relapsed Malignant Mesothelioma (CONFIRM): A Multicentre, Double-Blind, Randomised, Phase 3 Trial. Lancet Oncol..

[16] Peters S., Scherpereel A., Cornelissen R., Oulkhouir Y., Greillier L., Kaplan M.A., Talbot T., Monnet I., Hiret S., Baas P. (2022). First-Line Nivolumab plus Ipilimumab versus Chemotherapy in Patients with Unresectable Malignant Pleural Mesothelioma: 3-Year Outcomes from CheckMate 743. Ann. Oncol..

[17] Bylicki O., Guisier F., Scherpereel A., Daniel C., Swalduz A., Grolleau E., Bernardi M., Hominal S., Prevost J.B., Pamart G. (2024). Real-World Efficacy and Safety of Combination Nivolumab plus Ipilimumab for Untreated, Unresectable, Pleural Mesothelioma: The Meso-Immune (GFPC 04-2021) Trial. Lung Cancer Amst. Neth..

[18] Enrico D., Gomez J.E., Aguirre D., Tissera N.S., Tsou F., Pupareli C., Tanco D.P., Waisberg F., Rodríguez A., Rizzo M. (2024). Efficacy of First-Line Nivolumab Plus Ipilimumab in Unresectable Pleural Mesothelioma: A Multicenter Real-World Study (ImmunoMeso LATAM). Clin. Lung Cancer.

[19] Schmid S., Holer L., Gysel K., Koster K.-L., Rothschild S.I., Boos L.A., Frehner L., Cardoso Almeida S., Britschgi C., Metaxas Y. (2024). Real-World Outcomes of Patients With Malignant Pleural Mesothelioma Receiving a Combination of Ipilimumab and Nivolumab as First- or Later-Line Treatment. JTO Clin. Res. Rep..

[20] McNamee N., Harvey C., Gray L., Khoo T., Lingam L., Zhang B., Nindra U., Yip P.Y., Pal A., Clay T. (2024). Brief Report: Real-World Toxicity and Survival of Combination Immunotherapy in Pleural Mesothelioma—RIOMeso. J. Thorac. Oncol..

[21] Chow K.V.C., Turner C., Hughes B., Lwin Z., Chan B. (2024). Real-World Outcomes for Patients with Pleural Mesothelioma: A Multisite Retrospective Cohort Study. Asia Pac. J. Clin. Oncol..

[22] Dumoulin D.W., Douma L.H., Hofman M.M., van der Noort V., Cornelissen R., de Gooijer C.J., Burgers J.A., Aerts J.G.J.V. (2024). Nivolumab and Ipilimumab in the Real-World Setting in Patients with Mesothelioma. Lung Cancer Amst. Neth..

[23] Imai H. (2025). Current Drug Therapy for Pleural Mesothelioma. Respir. Investig..

[24] Sofianidi A.A., Syrigos N.K., Blyth K.G., Charpidou A., Vathiotis I.A. (2025). Breaking Through: Immunotherapy Innovations in Pleural Mesothelioma. Clin. Lung Cancer.

[25] Nowak A.K., Lesterhuis W.J., Kok P.-S., Brown C., Hughes B.G., Karikios D.J., John T., Kao S.C.-H., Leslie C., Cook A.M. (2020). Durvalumab with First-Line Chemotherapy in Previously Untreated Malignant Pleural Mesothelioma (DREAM): A Multicentre, Single-Arm, Phase 2 Trial with a Safety Run-In. Lancet Oncol..

[26] Forde P.M., Anagnostou V., Sun Z., Dahlberg S.E., Kindler H.L., Niknafs N., Purcell T., Santana-Davila R., Dudek A.Z., Borghaei H. (2021). Durvalumab with Platinum-Pemetrexed for Unresectable Pleural Mesothelioma: Survival, Genomic and Immunologic Analyses from the Phase 2 PrE0505 Trial. Nat. Med..

[27] Sauter J.L., Dacic S., Galateau-Salle F., Attanoos R.L., Butnor K.J., Churg A., Husain A.N., Kadota K., Khoor A., Nicholson A.G. (2022). The 2021 WHO Classification of Tumors of the Pleura: Advances Since the 2015 Classification. J. Thorac. Oncol..

[28] Calabrò L., Bronte G., Grosso F., Cerbone L., Delmonte A., Nicolini F., Mazza M., Di Giacomo A.M., Covre A., Lofiego M.F. (2023). Immunotherapy of Mesothelioma: The Evolving Change of a Long-Standing Therapeutic Dream. Front. Immunol..

[29] Dacic S. (2022). Pleural Mesothelioma Classification-Update and Challenges. Mod. Pathol..

[30] Bueno R., Stawiski E.W., Goldstein L.D., Durinck S., De Rienzo A., Modrusan Z., Gnad F., Nguyen T.T., Jaiswal B.S., Chirieac L.R. (2016). Comprehensive Genomic Analysis of Malignant Pleural Mesothelioma Identifies Recurrent Mutations, Gene Fusions and Splicing Alterations. Nat. Genet..

[31] Blum Y., Meiller C., Quetel L., Elarouci N., Ayadi M., Tashtanbaeva D., Armenoult L., Montagne F., Tranchant R., Renier A. (2019). Dissecting Heterogeneity in Malignant Pleural Mesothelioma through Histo-Molecular Gradients for Clinical Applications. Nat. Commun..

[32] Zhang M., Bzura A., Baitei E.Y., Zhou Z., Spicer J.B., Poile C., Rogel J., Branson A., King A., Barber S. (2024). A Gut Microbiota Rheostat Forecasts Responsiveness to PD-L1 and VEGF Blockade in Mesothelioma. Nat. Commun..

[33] Hmeljak J., Sanchez-Vega F., Hoadley K.A., Shih J., Stewart C., Heiman D., Tarpey P., Danilova L., Drill E., Gibb E.A. (2018). Integrative Molecular Characterization of Malignant Pleural Mesothelioma. Cancer Discov..

[34] Dagogo-Jack I., Madison R.W., Lennerz J.K., Chen K.-T., Hopkins J.F., Schrock A.B., Ritterhouse L.L., Lester A., Wharton K.A., Mino-Kenudson M. (2022). Molecular Characterization of Mesothelioma: Impact of Histologic Type and Site of Origin on Molecular Landscape. JCO Precis. Oncol..

[35] Xu D., Gao Y., Yang H., Spils M., Marti T.M., Losmanová T., Su M., Wang W., Zhou Q., Dorn P. (2024). BAP1 Deficiency Inflames the Tumor Immune Microenvironment and Is a Candidate Biomarker for Immunotherapy Response in Malignant Pleural Mesothelioma. JTO Clin. Res. Rep..

[36] Nash A., Creaney J. (2023). Genomic Landscape of Pleural Mesothelioma and Therapeutic Aftermaths. Curr. Oncol. Rep..

[37] Meiller C., Montagne F., Hirsch T.Z., Caruso S., de Wolf J., Bayard Q., Assié J.-B., Meunier L., Blum Y., Quetel L. (2021). Multi-Site Tumor Sampling Highlights Molecular Intra-Tumor Heterogeneity in Malignant Pleural Mesothelioma. Genome Med..

[38] Quetel L., Meiller C., Assié J.-B., Blum Y., Imbeaud S., Montagne F., Tranchant R., de Wolf J., Caruso S., Copin M.-C. (2020). Genetic Alterations of Malignant Pleural Mesothelioma: Association with Tumor Heterogeneity and Overall Survival. Mol. Oncol..

[39] Jin L., Gu W., Li X., Xie L., Wang L., Chen Z. (2020). PD-L1 and Prognosis in Patients with Malignant Pleural Mesothelioma: A Meta-Analysis and Bioinformatics Study. Ther. Adv. Med. Oncol..

[40] Brosseau S., Danel C., Scherpereel A., Mazières J., Lantuejoul S., Margery J., Greillier L., Audigier-Valette C., Gounant V., Antoine M. (2019). Shorter Survival in Malignant Pleural Mesothelioma Patients with High PD-L1 Expression Associated with Sarcomatoid or Biphasic Histology Subtype: A Series of 214 Cases From the Bio-MAPS Cohort. Clin. Lung Cancer.

[41] Pasello G., Zago G., Lunardi F., Urso L., Kern I., Vlacic G., Grosso F., Mencoboni M., Ceresoli G.L., Schiavon M. (2018). Malignant Pleural Mesothelioma Immune Microenvironment and Checkpoint Expression: Correlation with Clinical-Pathological Features and Intratumor Heterogeneity over Time. Ann. Oncol..

[42] Giotti B., Dolasia K., Zhao W., Cai P., Sweeney R., Merritt E., Kiner E., Kim G.S., Bhagwat A., Nguyen T. (2024). Single-Cell View of Tumor Microenvironment Gradients in Pleural Mesothelioma. Cancer Discov..

[43] Bertuccio F.R., Montini S., Fusco M.A., Di Gennaro A., Sciandrone G., Agustoni F., Galli G., Bortolotto C., Saddi J., Baietto G. (2025). Malignant Pleural Mesothelioma: From Pathophysiology to Innovative Actionable Targets. Cancers.

[44] Zaleski M., Kalhor N., Fujimoto J., Wistuba I., Moran C.A. (2020). Sarcomatoid Mesothelioma: A CDKN2A Molecular Analysis of 53 Cases with Immunohistochemical Correlation with BAP1. Pathol. Res. Pract..

[45] Baumann F., Flores E., Napolitano A., Kanodia S., Taioli E., Pass H., Yang H., Carbone M. (2015). Mesothelioma Patients with Germline BAP1 Mutations Have 7-Fold Improved Long-Term Survival. Carcinogenesis.

[46] Pastorino S., Yoshikawa Y., Pass H.I., Emi M., Nasu M., Pagano I., Takinishi Y., Yamamoto R., Minaai M., Hashimoto-Tamaoki T. (2018). A Subset of Mesotheliomas with Improved Survival Occurring in Carriers of BAP1 and Other Germline Mutations. J. Clin. Oncol..

[47] Cakiroglu E., Senturk S. (2020). Genomics and Functional Genomics of Malignant Pleural Mesothelioma. Int. J. Mol. Sci..

[48] Zhang N., Zhao Z., Long J., Li H., Zhang B., Chen G., Li X., Lv T., Zhang W., Ou X. (2017). Molecular Alterations of the NF2 Gene in Hepatocellular Carcinoma and Intrahepatic Cholangiocarcinoma. Oncol. Rep..

[49] Elsayed A.M., Kittaneh M., Cebulla C.M., Abdel-Rahman M.H. (2025). An Overview of BAP1 Biological Functions and Current Therapeutics. Biochim. Biophys. Acta Rev. Cancer.

[50] Nardone V., Porta C., Giannicola R., Correale P., Mutti L. (2022). Abemaciclib for Malignant Pleural Mesothelioma. Lancet Oncol..

[51] Zhang M., Luo J.-L., Sun Q., Harber J., Dawson A.G., Nakas A., Busacca S., Sharkey A.J., Waller D., Sheaff M.T. (2021). Clonal Architecture in Mesothelioma Is Prognostic and Shapes the Tumour Microenvironment. Nat. Commun..

[52] Torricelli F., Spada F., Bishop C., Todd K., Nonaka D., Petrov N., Barberio M.T., Ramsay A.G., Ellis R., Ciarrocchi A. (2025). The Phenogenomic Landscapes of Pleural Mesothelioma Tumor Microenvironment Predict Clinical Outcomes. J. Transl. Med..

[53] Marcus L., Fashoyin-Aje L.A., Donoghue M., Yuan M., Rodriguez L., Gallagher P.S., Philip R., Ghosh S., Theoret M.R., Beaver J.A. (2021). FDA Approval Summary: Pembrolizumab for the Treatment of Tumor Mutational Burden-High Solid Tumors. Clin. Cancer Res..

[54] Mansfield A.S., Peikert T., Vasmatzis G. (2020). Chromosomal Rearrangements and Their Neoantigenic Potential in Mesothelioma. Transl. Lung Cancer Res..

[55] Davis A.A., Patel V.G. (2019). The Role of PD-L1 Expression as a Predictive Biomarker: An Analysis of All US Food and Drug Administration (FDA) Approvals of Immune Checkpoint Inhibitors. J. Immunother. Cancer.

[56] Chu Q., Perrone F., Greillier L., Tu W., Piccirillo M.C., Grosso F., Lo Russo G., Florescu M., Mencoboni M., Morabito A. (2023). Pembrolizumab plus Chemotherapy versus Chemotherapy in Untreated Advanced Pleural Mesothelioma in Canada, Italy, and France: A Phase 3, Open-Label, Randomised Controlled Trial. Lancet Lond. Engl..

[57] Tagliamento M., Bironzo P., Curcio H., De Luca E., Pignataro D., Rapetti S.G., Audisio M., Bertaglia V., Paratore C., Bungaro M. (2022). A Systematic Review and Meta-Analysis of Trials Assessing PD-1/PD-L1 Immune Checkpoint Inhibitors Activity in Pre-Treated Advanced Stage Malignant Mesothelioma. Crit. Rev. Oncol. Hematol..

[58] Homicsko K., Zygoura P., Norkin M., Tissot S., Shakarishvili N., Popat S., Curioni-Fontecedro A., O’Brien M., Pope A., Shah R. (2023). PD-1-Expressing Macrophages and CD8 T Cells Are Independent Predictors of Clinical Benefit from PD-1 Inhibition in Advanced Mesothelioma. J. Immunother. Cancer.

[59] Marcq E., Waele J.D., Audenaerde J.V., Lion E., Santermans E., Hens N., Pauwels P., van Meerbeeck J.P., Smits E.L.J. (2017). Abundant Expression of TIM-3, LAG-3, PD-1 and PD-L1 as Immunotherapy Checkpoint Targets in Effusions of Mesothelioma Patients. Oncotarget.

[60] Cedres S., Valdivia A., Priano I., Rocha P., Iranzo P., Pardo N., Martinez-Marti A., Felip E. (2025). BAP1 Mutations and Pleural Mesothelioma: Genetic Insights, Clinical Implications, and Therapeutic Perspectives. Cancers.

[61] Yang H., Xu D., Gao Y., Schmid R.A., Peng R.-W. (2020). Oncolytic Viral Therapy for Malignant Pleural Mesothelioma. J. Thorac. Oncol..

[62] Jacquelot N., Yamazaki T., Roberti M.P., Duong C.P.M., Andrews M.C., Verlingue L., Ferrere G., Becharef S., Vétizou M., Daillère R. (2019). Sustained Type I Interferon Signaling as a Mechanism of Resistance to PD-1 Blockade. Cell Res..

[63] Shrestha R., Nabavi N., Lin Y.-Y., Mo F., Anderson S., Volik S., Adomat H.H., Lin D., Xue H., Dong X. (2019). BAP1 Haploinsufficiency Predicts a Distinct Immunogenic Class of Malignant Peritoneal Mesothelioma. Genome Med..

[64] Ogbue O., Unlu S., Sorathia S., Nanah A.R., Abdeljaleel F., Haddad A.S., Daw H. (2023). Impact of BAP1 Mutational Status on Immunotherapy Outcomes in Advanced Malignant Pleural Mesothelioma: A Single Institution Experience. J. Clin. Oncol..

[65] Fennell D.A., King A., Mohammed S., Greystoke A., Anthony S., Poile C., Nusrat N., Scotland M., Bhundia V., Branson A. (2022). Abemaciclib in Patients with p16ink4A-Deficient Mesothelioma (MiST2): A Single-Arm, Open-Label, Phase 2 Trial. Lancet Oncol..

[66] Henrich F.C., Singer K., Poller K., Bernhardt L., Strobl C.D., Limm K., Ritter A.P., Gottfried E., Völkl S., Jacobs B. (2016). Suppressive Effects of Tumor Cell-Derived 5′-Deoxy-5′-Methylthioadenosine on Human T Cells. Oncoimmunology.

[67] Chan J.M.-L., Chang Y.-C., Chan H.-C., Chan H.-C., Chang W.-C., Wang L.-F., Tsai T.-H., Chen Y.-J., Huang W.-C. (2024). FK228 Suppress the Growth of Human Malignant Pleural Mesothelioma Tumor Independent to Epithelioid or Non-Epithelioid Histology. Mol. Med. Camb. Mass.

[68] Janse van Rensburg H.J., Azad T., Ling M., Hao Y., Snetsinger B., Khanal P., Minassian L.M., Graham C.H., Rauh M.J., Yang X. (2018). The Hippo Pathway Component TAZ Promotes Immune Evasion in Human Cancer through PD-L1. Cancer Res..

[69] Kim M.H., Kim C.G., Kim S.-K., Shin S.J., Choe E.A., Park S.-H., Shin E.-C., Kim J. (2018). YAP-Induced PD-L1 Expression Drives Immune Evasion in BRAFi-Resistant Melanoma. Cancer Immunol. Res..

[70] Minnema-Luiting J., Vroman H., Aerts J., Cornelissen R. (2018). Heterogeneity in Immune Cell Content in Malignant Pleural Mesothelioma. Int. J. Mol. Sci..

[71] Hiltbrunner S., Mannarino L., Kirschner M.B., Opitz I., Rigutto A., Laure A., Lia M., Nozza P., Maconi A., Marchini S. (2021). Tumor Immune Microenvironment and Genetic Alterations in Mesothelioma. Front. Oncol..

[72] Galon J., Bruni D. (2019). Approaches to Treat Immune Hot, Altered and Cold Tumours with Combination Immunotherapies. Nat. Rev. Drug Discov..

[73] Jing H., Gao Y., Sun Z., Liu S. (2025). Recent Advances in Novel Tumor Immunotherapy Strategies Based on Regulating the Tumor Microenvironment and Immune Checkpoints. Front. Immunol..

[74] Alay A., Cordero D., Hijazo-Pechero S., Aliagas E., Lopez-Doriga A., Marín R., Palmero R., Llatjós R., Escobar I., Ramos R. (2021). Integrative Transcriptome Analysis of Malignant Pleural Mesothelioma Reveals a Clinically Relevant Immune-Based Classification. J. Immunother. Cancer.

[75] Chee S.J., Lopez M., Mellows T., Gankande S., Moutasim K.A., Harris S., Clarke J., Vijayanand P., Thomas G.J., Ottensmeier C.H. (2017). Evaluating the Effect of Immune Cells on the Outcome of Patients with Mesothelioma. Br. J. Cancer.

[76] Mannarino L., Paracchini L., Pezzuto F., Olteanu G.E., Moracci L., Vedovelli L., De Simone I., Bosetti C., Lupi M., Amodeo R. (2022). Epithelioid Pleural Mesothelioma Is Characterized by Tertiary Lymphoid Structures in Long Survivors: Results from the MATCH Study. Int. J. Mol. Sci..

[77] Cui X., Gu X., Li D., Wu P., Sun N., Zhang C., He J. (2025). Tertiary Lymphoid Structures as a Biomarker in Immunotherapy and beyond: Advancing towards Clinical Application. Cancer Lett..

[78] Cornelissen R., Lievense L.A., Maat A.P., Hendriks R.W., Hoogsteden H.C., Bogers A.J., Hegmans J.P., Aerts J.G. (2014). Ratio of Intratumoral Macrophage Phenotypes Is a Prognostic Factor in Epithelioid Malignant Pleural Mesothelioma. PLoS ONE.

[79] Horio D., Minami T., Kitai H., Ishigaki H., Higashiguchi Y., Kondo N., Hirota S., Kitajima K., Nakajima Y., Koda Y. (2020). Tumor-Associated Macrophage-Derived Inflammatory Cytokine Enhances Malignant Potential of Malignant Pleural Mesothelioma. Cancer Sci..

[80] Yang K., Yang T., Yang T., Yuan Y., Li F. (2022). Unraveling Tumor Microenvironment Heterogeneity in Malignant Pleural Mesothelioma Identifies Biologically Distinct Immune Subtypes Enabling Prognosis Determination. Front. Oncol..

[81] Ollila H., Mäyränpää M.I., Paavolainen L., Paajanen J., Välimäki K., Sutinen E., Wolff H., Räsänen J., Kallioniemi O., Myllärniemi M. (2022). Prognostic Role of Tumor Immune Microenvironment in Pleural Epithelioid Mesothelioma. Front. Oncol..

[82] Harber J., Kamata T., Pritchard C., Fennell D. (2021). Matter of TIME: The Tumor-Immune Microenvironment of Mesothelioma and Implications for Checkpoint Blockade Efficacy. J. Immunother. Cancer.

[83] Fridman W.H., Zitvogel L., Sautès-Fridman C., Kroemer G. (2017). The Immune Contexture in Cancer Prognosis and Treatment. Nat. Rev. Clin. Oncol..

[84] Magkouta S.F., Vaitsi P.C., Pappas A.G., Iliopoulou M., Kosti C.N., Psarra K., Kalomenidis I.T. (2021). CSF1/CSF1R Axis Blockade Limits Mesothelioma and Enhances Efficiency of Anti-PDL1 Immunotherapy. Cancers.

[85] Ibrahim A., Mohamady Farouk Abdalsalam N., Liang Z., Kashaf Tariq H., Li R., O. Afolabi L., Rabiu L., Chen X., Xu S., Xu Z. (2025). MDSC Checkpoint Blockade Therapy: A New Breakthrough Point Overcoming Immunosuppression in Cancer Immunotherapy. Cancer Gene Ther..

[86] Salaroglio I.C., Kopecka J., Napoli F., Pradotto M., Maletta F., Costardi L., Gagliasso M., Milosevic V., Ananthanarayanan P., Bironzo P. (2019). Potential Diagnostic and Prognostic Role of Microenvironment in Malignant Pleural Mesothelioma. J. Thorac. Oncol..

[87] Pathria P., Louis T.L., Varner J.A. (2019). Targeting Tumor-Associated Macrophages in Cancer. Trends Immunol..

[88] Xiao Z., Todd L., Huang L., Noguera-Ortega E., Lu Z., Huang L., Kopp M., Li Y., Pattada N., Zhong W. (2023). Desmoplastic Stroma Restricts T Cell Extravasation and Mediates Immune Exclusion and Immunosuppression in Solid Tumors. Nat. Commun..

[89] Chakravarthy A., Khan L., Bensler N.P., Bose P., De Carvalho D.D. (2018). TGF-β-Associated Extracellular Matrix Genes Link Cancer-Associated Fibroblasts to Immune Evasion and Immunotherapy Failure. Nat. Commun..

[90] Zhang T., Ren Y., Yang P., Wang J., Zhou H. (2022). Cancer-Associated Fibroblasts in Pancreatic Ductal Adenocarcinoma. Cell Death Dis..

[91] Grauel A.L., Nguyen B., Ruddy D., Laszewski T., Schwartz S., Chang J., Chen J., Piquet M., Pelletier M., Yan Z. (2020). TGFβ-Blockade Uncovers Stromal Plasticity in Tumors by Revealing the Existence of a Subset of Interferon-Licensed Fibroblasts. Nat. Commun..

[92] Qian B.-Z., Pollard J.W. (2010). Macrophage Diversity Enhances Tumor Progression and Metastasis. Cell.

[93] Creaney J., Patch A.-M., Addala V., Sneddon S.A., Nones K., Dick I.M., Lee Y.C.G., Newell F., Rouse E.J., Naeini M.M. (2022). Comprehensive Genomic and Tumour Immune Profiling Reveals Potential Therapeutic Targets in Malignant Pleural Mesothelioma. Genome Med..

[94] Torricelli F., Donati B., Reggiani F., Manicardi V., Piana S., Valli R., Lococo F., Ciarrocchi A. (2023). Spatially Resolved, High-Dimensional Transcriptomics Sorts out the Evolution of Biphasic Malignant Pleural Mesothelioma: New Paradigms for Immunotherapy. Mol. Cancer.

[95] An H.J., Chon H.J., Kim C. (2021). Peripheral Blood-Based Biomarkers for Immune Checkpoint Inhibitors. Int. J. Mol. Sci..

[96] Han S., Georgiev P., Ringel A.E., Sharpe A.H., Haigis M.C. (2023). Age-Associated Remodeling of T Cell Immunity and Metabolism. Cell Metab..

[97] Rodriguez J.E., Naigeon M., Goldschmidt V., Roulleaux Dugage M., Seknazi L., Danlos F.X., Champiat S., Marabelle A., Michot J.-M., Massard C. (2022). Immunosenescence, Inflammaging, and Cancer Immunotherapy Efficacy. Expert Rev. Anticancer Ther..

[98] Park M.D., Le Berichel J., Hamon P., Wilk C.M., Belabed M., Yatim N., Saffon A., Boumelha J., Falcomatà C., Tepper A. (2024). Hematopoietic Aging Promotes Cancer by Fueling IL-1α-Driven Emergency Myelopoiesis. Science.

[99] Bleve A., Consonni F.M., Porta C., Garlatti V., Sica A. (2022). Evolution and Targeting of Myeloid Suppressor Cells in Cancer: A Translational Perspective. Cancers.

[100] Peranzoni E., Ingangi V., Masetto E., Pinton L., Marigo I. (2020). Myeloid Cells as Clinical Biomarkers for Immune Checkpoint Blockade. Front. Immunol..

[101] Chen N., Liu S., Huang L., Li W., Yang W., Cong T., Ding L., Qiu M. (2017). Prognostic Significance of Neutrophil-to-Lymphocyte Ratio in Patients with Malignant Pleural Mesothelioma: A Meta-Analysis. Oncotarget.

[102] Okita R., Kawamoto N., Okada M., Inokawa H., Yamamoto N., Murakami T., Ikeda E. (2023). Preoperative Neutrophil-to-Lymphocyte Ratio Correlates with PD-L1 Expression in Immune Cells of Patients with Malignant Pleural Mesothelioma and Predicts Prognosis. Sci. Rep..

[103] Kazandjian D., Gong Y., Keegan P., Pazdur R., Blumenthal G.M. (2019). Prognostic Value of the Lung Immune Prognostic Index for Patients Treated for Metastatic Non-Small Cell Lung Cancer. JAMA Oncol..

[104] Mezquita L., Auclin E., Ferrara R., Charrier M., Remon J., Planchard D., Ponce S., Ares L.P., Leroy L., Audigier-Valette C. (2018). Association of the Lung Immune Prognostic Index with Immune Checkpoint Inhibitor Outcomes in Patients with Advanced Non-Small Cell Lung Cancer. JAMA Oncol..

[105] Guo Y., Pan Y., Wan J., Gong B., Li Y., Kan X., Zheng C. (2024). Prognosis Stratification of Cancer Patients Treated with Immune Checkpoint Inhibitors through Lung Immune Prognostic Index: A Meta-Analysis and Systematic Review. BMC Cancer.

[106] Liu J., Chen J., Xie C., Chen H., Yang S., Jiang H., Xu Y. (2025). The Predictive Significance of Various Prognostic Scoring Systems on the Efficacy of Immunotherapy in Non-Small Cell Lung Cancer Patients: A Retrospective Study. Health Sci. Rep..

[107] Pierro M., Baldini C., Auclin E., Vincent H., Varga A., Martin Romano P., Vuagnat P., Besse B., Planchard D., Hollebecque A. (2022). Predicting Immunotherapy Outcomes in Older Patients with Solid Tumors Using the LIPI Score. Cancers.

[108] Willems M., Scherpereel A., Wasielewski E., Raskin J., Brossel H., Fontaine A., Grégoire M., Halkin L., Jamakhani M., Heinen V. (2023). Excess of Blood Eosinophils Prior to Therapy Correlates with Worse Prognosis in Mesothelioma. Front. Immunol..

[109] Grisaru-Tal S., Rothenberg M.E., Munitz A. (2022). Eosinophil–Lymphocyte Interactions in the Tumor Microenvironment and Cancer Immunotherapy. Nat. Immunol..

[110] Elkrief A., Pidgeon R., Maleki Vareki S., Messaoudene M., Castagner B., Routy B. (2025). The Gut Microbiome as a Target in Cancer Immunotherapy: Opportunities and Challenges for Drug Development. Nat. Rev. Drug Discov..

[111] Pentimalli F., Krstic-Demonacos M., Costa C., Mutti L., Bakker E.Y. (2023). Intratumor Microbiota as a Novel Potential Prognostic Indicator in Mesothelioma. Front. Immunol..

[112] Kwok B., Wu B.G., Kocak I.F., Sulaiman I., Schluger R., Li Y., Anwer R., Goparaju C., Ryan D.J., Sagatelian M. (2023). Pleural Fluid Microbiota as a Biomarker for Malignancy and Prognosis. Sci. Rep..

[113] Paik J. (2022). Nivolumab Plus Relatlimab: First Approval. Drugs.

[114] Shi H., Yu T.-K., Johnson B., Selvamani S.P., Zhuang L., Lee K., Klebe S., Smith S., Wong K., Chen K. (2025). A Combination of PD-1 and TIGIT Immune Checkpoint Inhibitors Elicits a Strong Anti-Tumour Response in Mesothelioma. J. Exp. Clin. Cancer Res..

[115] Rovers S., Van Audenaerde J., Verloy R., De Waele J., Brants L., Hermans C., Lau H.W., Merlin C., Möller Ribas M., Ponsaerts P. (2025). Co-Targeting of VEGFR2 and PD-L1 Promotes Survival and Vasculature Normalization in Pleural Mesothelioma. OncoImmunology.

[116] Garofalo M., Wieczorek M., Anders I., Staniszewska M., Lazniewski M., Prygiel M., Zasada A.A., Szczepińska T., Plewczynski D., Salmaso S. (2023). Novel Combinatorial Therapy of Oncolytic Adenovirus AdV5/3-D24-ICOSL-CD40L with Anti PD-1 Exhibits Enhanced Anti-Cancer Efficacy through Promotion of Intratumoral T-Cell Infiltration and Modulation of Tumour Microenvironment in Mesothelioma Mouse Model. Front. Oncol..

[117] D’Alonzo R.A., Keam S., Gill S., Rowshanfarzad P., Nowak A.K., Ebert M.A., Cook A.M. (2025). Fractionated Low-Dose Radiotherapy Primes the Tumor Microenvironment for Immunotherapy in a Murine Mesothelioma Model. Cancer Immunol. Immunother. CII.

[118] Miyamoto Y., Kozuki T., Aoe K., Wada S., Harada D., Yoshida M., Sakurai J., Hotta K., Fujimoto N. (2021). JME-001 Phase II Trial of First-Line Combination Chemotherapy with Cisplatin, Pemetrexed, and Nivolumab for Unresectable Malignant Pleural Mesothelioma. J. Immunother. Cancer.

[119] Kok P.S., Forde P.M., Hughes B., Sun Z., Brown C., Ramalingam S., Cook A., Lesterhuis W.J., Yip S., O’Byrne K. (2022). Protocol of DREAM3R: DuRvalumab with chEmotherapy as First-Line treAtment in Advanced Pleural Mesothelioma-a Phase 3 Randomised Trial. BMJ Open.

[120] Felip E., Popat S., Dafni U., Ribi K., Pope A., Cedres S., Shah R., de Marinis F., Cove Smith L., Bernabé R. (2025). A Randomised Phase III Study of Bevacizumab and Carboplatin-Pemetrexed Chemotherapy with or without Atezolizumab as First-Line Treatment for Advanced Pleural Mesothelioma: Results of the ETOP 13-18 BEAT-Meso Trial. Ann. Oncol..

[121] Frentzas S., Kao S., Gao R., Zheng H., Rizwan A., Budha N., Pedroza L.d.l.H., Tan W., Meniawy T. (2023). AdvanTIG-105: A Phase I Dose Escalation Study of the Anti-TIGIT Monoclonal Antibody Ociperlimab in Combination with Tislelizumab in Patients with Advanced Solid Tumors. J. Immunother. Cancer.

[122] Dudek A.Z., Xi M.X., Scilla K.A., Mamdani H., Creelan B.C., Saltos A., Tanvetyanon T., Chiappori A. (2023). Phase 2 Trial of Nivolumab and Ramucirumab for Relapsed Mesothelioma: HCRN-LUN15-299. JTO Clin. Res. Rep..

[123] Douma L.-A.H., van der Noort V., Lalezari F., de Vries J.F., Monkhorst K., Smesseim I., Baas P., Schilder B., Vermeulen M., Burgers J.A. (2025). Pembrolizumab plus Lenvatinib as Second-Line Treatment in Patients with Pleural Mesothelioma (PEMMELA): Cohort 2 of a Single-Arm, Phase 2 Study. Lancet Oncol..

[124] Papadatos-Pastos D., Yuan W., Pal A., Crespo M., Ferreira A., Gurel B., Prout T., Ameratunga M., Chénard-Poirier M., Curcean A. (2022). Phase 1, Dose-Escalation Study of Guadecitabine (SGI-110) in Combination with Pembrolizumab in Patients with Solid Tumors. J. Immunother. Cancer.

[125] (2018). NHS Greater Glasgow and Clyde A Phase I/IIA Study to Assess Safety, Tolerability and Preliminary Activity of the Combination of FAK (Defactinib) and PD-1 (Pembrolizumab) Inhibition in Patients with Advanced Solid Malignancies (FAK-PD1). https://clin.larvol.com/trial-detail/2015-003928-31.

[126] van Gulijk M., Belderbos B., Dumoulin D., Cornelissen R., Bezemer K., Klaase L., Dammeijer F., Aerts J. (2023). Combination of PD-1/PD-L1 Checkpoint Inhibition and Dendritic Cell Therapy in Mice Models and in Patients with Mesothelioma. Int. J. Cancer.

[127] Haakensen V.D., Öjlert Å.K., Thunold S., Farooqi S., Nowak A.K., Chin W.L., Grundberg O., Szejniuk W.M., Cedres S., Sørensen J.B. (2024). UV1 Telomerase Vaccine with Ipilimumab and Nivolumab as Second Line Treatment for Pleural Mesothelioma—A Phase II Randomised Trial. Eur. J. Cancer.

[128] Adusumilli P.S., Zauderer M.G., Rivière I., Solomon S.B., Rusch V.W., O’Cearbhaill R.E., Zhu A., Cheema W., Chintala N.K., Halton E. (2021). A Phase I Trial of Regional Mesothelin-Targeted CAR T-Cell Therapy in Patients with Malignant Pleural Disease, in Combination with the Anti-PD-1 Agent Pembrolizumab. Cancer Discov..

[129] Mampuya W.A., Bouchaab H., Schaefer N., Kinj R., La Rosa S., Letovanec I., Ozsahin M., Bourhis J., Coukos G., Peters S. (2021). Abscopal Effect in a Patient with Malignant Pleural Mesothelioma Treated with Palliative Radiotherapy and Pembrolizumab. Clin. Transl. Radiat. Oncol..

[130] Rittberg R., Chan E., Yip S., Alex D., Ho C. (2022). Radiation Induced Abscopal Effect in a Patient with Malignant Pleural Mesothelioma on Pembrolizumab. Cureus.

[131] Lee H.-S., Jang H.-J., Ramineni M., Wang D.Y., Ramos D., Choi J.M., Splawn T., Espinoza M., Almarez M., Hosey L. (2023). A Phase II Window of Opportunity Study of Neoadjuvant PD-L1 versus PD-L1 plus CTLA-4 Blockade for Patients with Malignant Pleural Mesothelioma. Clin. Cancer Res..

[132] Reuss J.E., Lee P.K., Mehran R.J., Hu C., Ke S., Jamali A., Najjar M., Niknafs N., Wehr J., Oner E. (2025). Perioperative Nivolumab or Nivolumab plus Ipilimumab in Resectable Diffuse Pleural Mesothelioma: A Phase 2 Trial and ctDNA Analyses. Nat. Med..

[133] Catanzaro E., Beltrán-Visiedo M., Galluzzi L., Krysko D.V. (2025). Immunogenicity of Cell Death and Cancer Immunotherapy with Immune Checkpoint Inhibitors. Cell. Mol. Immunol..

[134] Zitvogel L., Apetoh L., Ghiringhelli F., Kroemer G. (2008). Immunological Aspects of Cancer Chemotherapy. Nat. Rev. Immunol..

[135] Kroemer G., Galassi C., Zitvogel L., Galluzzi L. (2022). Immunogenic Cell Stress and Death. Nat. Immunol..

[136] Galluzzi L., Guilbaud E., Schmidt D., Kroemer G., Marincola F.M. (2024). Targeting Immunogenic Cell Stress and Death for Cancer Therapy. Nat. Rev. Drug Discov..

[137] Zalcman G., Mazieres J., Margery J., Greillier L., Audigier-Valette C., Moro-Sibilot D., Molinier O., Corre R., Monnet I., Gounant V. (2016). Bevacizumab for Newly Diagnosed Pleural Mesothelioma in the Mesothelioma Avastin Cisplatin Pemetrexed Study (MAPS): A Randomised, Controlled, Open-Label, Phase 3 Trial. Lancet Lond. Engl..

[138] Scagliotti G.V., Gaafar R., Nowak A.K., Nakano T., van Meerbeeck J., Popat S., Vogelzang N.J., Grosso F., Aboelhassan R., Jakopovic M. (2019). Nintedanib in Combination with Pemetrexed and Cisplatin for Chemotherapy-Naive Patients with Advanced Malignant Pleural Mesothelioma (LUME-Meso): A Double-Blind, Randomised, Placebo-Controlled Phase 3 Trial. Lancet Respir. Med..

[139] Pinto C., Zucali P.A., Pagano M., Grosso F., Pasello G., Garassino M.C., Tiseo M., Soto Parra H., Grossi F., Cappuzzo F. (2021). Gemcitabine with or without Ramucirumab as Second-Line Treatment for Malignant Pleural Mesothelioma (RAMES): A Randomised, Double-Blind, Placebo-Controlled, Phase 2 Trial. Lancet Oncol..

[140] Arimura K., Hiroshima K., Nagashima Y., Nakazawa T., Ogihara A., Orimo M., Sato Y., Katsura H., Kanzaki M., Kondo M. (2023). LAG3 Is an Independent Prognostic Biomarker and Potential Target for Immune Checkpoint Inhibitors in Malignant Pleural Mesothelioma: A Retrospective Study. BMC Cancer.

[141] Lu C., Tan Y. (2024). Promising Immunotherapy Targets: TIM3, LAG3, and TIGIT Joined the Party. Mol. Ther. Oncol..

[142] Rovers S., Janssens A., Raskin J., Pauwels P., van Meerbeeck J.P., Smits E., Marcq E. (2022). Recent Advances of Immune Checkpoint Inhibition and Potential for (Combined) TIGIT Blockade as a New Strategy for Malignant Pleural Mesothelioma. Biomedicines.

[143] Gao Y., He Y., Tang Y., Chen Z.-S., Qu M. (2024). VISTA: A Novel Checkpoint for Cancer Immunotherapy. Drug Discov. Today.

[144] Noelle R.J., Lines J.L., Lewis L.D., Martell R.E., Guillaudeux T., Lee S.W., Mahoney K.M., Vesely M.D., Boyd-Kirkup J., Nambiar D.K. (2023). Clinical and Research Updates on the VISTA Immune Checkpoint: Immuno-Oncology Themes and Highlights. Front. Oncol..

[145] Goswami S., Anandhan S., Raychaudhuri D., Sharma P. (2023). Myeloid Cell-Targeted Therapies for Solid Tumours. Nat. Rev. Immunol..

[146] Blondy T., d’Almeida S.M., Briolay T., Tabiasco J., Meiller C., Chéné A.-L., Cellerin L., Deshayes S., Delneste Y., Fonteneau J.-F. (2020). Involvement of the M-CSF/IL-34/CSF-1R Pathway in Malignant Pleural Mesothelioma. J. Immunother. Cancer.

[147] Joalland N., Quéméner A., Deshayes S., Humeau R., Maillasson M., LeBihan H., Salama A., Fresquet J., Remy S., Mortier E. (2025). New Soluble CSF-1R-Dimeric Mutein with Enhanced Trapping of Both CSF-1 and IL-34 Reduces Suppressive Tumor-Associated Macrophages in Pleural Mesothelioma. J. Immunother. Cancer.

[148] Jian C.-Z., Lin L., Hsu C.-L., Chen Y.-H., Hsu C., Tan C.-T., Ou D.-L. (2024). A Potential Novel Cancer Immunotherapy: Agonistic Anti-CD40 Antibodies. Drug Discov. Today.

[149] McVey J.C., Beatty G.L. (2025). Facts and Hopes of CD40 Agonists in Cancer Immunotherapy. Clin. Cancer Res..

[150] Jackaman C., Cornwall S., Graham P.T., Nelson D.J. (2011). CD40-Activated B Cells Contribute to Mesothelioma Tumor Regression. Immunol. Cell Biol..

[151] Khong A., Brown M.D., Vivian J.B., Robinson B.W.S., Currie A.J. (2013). Agonistic Anti-CD40 Antibody Therapy Is Effective against Postoperative Cancer Recurrence and Metastasis in a Murine Tumor Model. J. Immunother..

[152] Pronk N.B., Polman A.J., Sterk L., Oosterhuis J.W.A., Smit E.F. (2009). A Nonresponding Small Cell Lung Carcinoma. J. Thorac. Oncol..

[153] Broomfield S., Most R., Prosser A., Mahendran S., Tovey M., Smyth M., Robinson B., Currie A. (2009). Locally Administered TLR7 Agonists Drive Systemic Antitumor Immune Responses That Are Enhanced by Anti-CD40 Immunotherapy. J. Immunol..

[154] Khong A., Cleaver A.L., Fahmi Alatas M., Wylie B.C., Connor T., Fisher S.A., Broomfield S., Lesterhuis W.J., Currie A.J., Lake R.A. (2014). The Efficacy of Tumor Debulking Surgery Is Improved by Adjuvant Immunotherapy Using Imiquimod and Anti-CD40. BMC Cancer.

[155] Jackaman C., Lansley S., Allan J.E., Robinson B.W.S., Nelson D.J. (2012). IL-2/CD40-Driven NK Cells Install and Maintain Potency in the Anti-Mesothelioma Effector/Memory Phase. Int. Immunol..

[156] Nowak A.K., Cook A.M., McDonnell A.M., Millward M.J., Creaney J., Francis R.J., Hasani A., Segal A., Musk A.W., Turlach B.A. (2015). A Phase 1b Clinical Trial of the CD40-Activating Antibody CP-870,893 in Combination with Cisplatin and Pemetrexed in Malignant Pleural Mesothelioma. Ann. Oncol..

[157] Luke J.J., Barlesi F., Chung K., Tolcher A.W., Kelly K., Hollebecque A., Le Tourneau C., Subbiah V., Tsai F., Kao S. (2021). Phase I Study of ABBV-428, a Mesothelin-CD40 Bispecific, in Patients with Advanced Solid Tumors. J. Immunother. Cancer.

[158] Gomez-Randulfe I., Lavender H., Symeonides S., Blackhall F. (2025). Impact of Systemic Anticancer Therapy Timing on Cancer Vaccine Immunogenicity: A Review. Ther. Adv. Med. Oncol..

[159] Cornelissen R., Hegmans J.P.J.J., Maat A.P.W.M., Kaijen-Lambers M.E.H., Bezemer K., Hendriks R.W., Hoogsteden H.C., Aerts J.G.J.V. (2016). Extended Tumor Control after Dendritic Cell Vaccination with Low-Dose Cyclophosphamide as Adjuvant Treatment in Patients with Malignant Pleural Mesothelioma. Am. J. Respir. Crit. Care Med..

[160] Noordam L., Kaijen M.E.H., Bezemer K., Cornelissen R., Maat L.A.P.W.M., Hoogsteden H.C., Aerts J.G.J.V., Hendriks R.W., Hegmans J.P.J.J., Vroman H. (2018). Low-Dose Cyclophosphamide Depletes Circulating Naïve and Activated Regulatory T Cells in Malignant Pleural Mesothelioma Patients Synergistically Treated with Dendritic Cell-Based Immunotherapy. OncoImmunology.

[161] Albiges L., Gurney H., Atduev V., Suarez C., Climent M.A., Pook D., Tomczak P., Barthelemy P., Lee J.L., Stus V. (2023). Pembrolizumab plus Lenvatinib as First-Line Therapy for Advanced Non-Clear-Cell Renal Cell Carcinoma (KEYNOTE-B61): A Single-Arm, Multicentre, Phase 2 Trial. Lancet Oncol..

[162] Lee D.U., Han B.S., Jung K.H., Hong S.-S. (2024). Tumor Stroma as a Therapeutic Target for Pancreatic Ductal Adenocarcinoma. Biomol. Ther..

[163] Sato N., Cheng X.-B., Kohi S., Koga A., Hirata K. (2016). Targeting Hyaluronan for the Treatment of Pancreatic Ductal Adenocarcinoma. Acta Pharm. Sin. B.

[164] Symeonides S.N., Anderton S.M., Serrels A. (2017). FAK-Inhibition Opens the Door to Checkpoint Immunotherapy in Pancreatic Cancer. J. Immunother. Cancer.

[165] Jiang H., Hegde S., Knolhoff B.L., Zhu Y., Herndon J.M., Meyer M.A., Nywening T.M., Hawkins W.G., Shapiro I.M., Weaver D.T. (2016). Targeting Focal Adhesion Kinase Renders Pancreatic Cancers Responsive to Checkpoint Immunotherapy. Nat. Med..

[166] Wang-Gillam A., Lim K.-H., McWilliams R., Suresh R., Lockhart A.C., Brown A., Breden M., Belle J.I., Herndon J., Bogner S.J. (2022). Defactinib, Pembrolizumab, and Gemcitabine in Patients with Advanced Treatment Refractory Pancreatic Cancer: A Phase I Dose Escalation and Expansion Study. Clin. Cancer Res..

[167] Quach H.T., Hou Z., Bellis R.Y., Saini J.K., Amador-Molina A., Adusumilli P.S., Xiong Y. (2022). Next-Generation Immunotherapy for Solid Tumors: Combination Immunotherapy with Crosstalk Blockade of TGFβ and PD-1/PD-L1. Expert Opin. Investig. Drugs.

[168] Stevenson J.P., Kindler H.L., Papasavvas E., Sun J., Jacobs-Small M., Hull J., Schwed D., Ranganathan A., Newick K., Heitjan D.F. (2013). Immunological Effects of the TGFβ-Blocking Antibody GC1008 in Malignant Pleural Mesothelioma Patients. OncoImmunology.

[169] Mohammad H.P., Barbash O., Creasy C.L. (2019). Targeting Epigenetic Modifications in Cancer Therapy: Erasing the Roadmap to Cancer. Nat. Med..

[170] Targeting the Epigenetic Regulation of Antitumour Immunity. 2020—Cerca Con Google. https://www.nature.com/articles/s41573-020-0077-5.

[171] Villanueva L., Álvarez-Errico D., Esteller M. (2020). The Contribution of Epigenetics to Cancer Immunotherapy. Trends Immunol..

[172] Duan R., Du W., Guo W. (2020). EZH2: A Novel Target for Cancer Treatment. J. Hematol. Oncol..

[173] Al Khatib M.O., Pinton G., Moro L., Porta C. (2023). Benefits and Challenges of Inhibiting EZH2 in Malignant Pleural Mesothelioma. Cancers.

[174] Sabour-Takanlou M., Sabour-Takanlou L., Biray-Avci C. (2024). EZH2-Associated Tumor Malignancy: A Prominent Target for Cancer Treatment. Clin. Genet..

[175] Zauderer M.G., Szlosarek P.W., Le Moulec S., Popat S., Taylor P., Planchard D., Scherpereel A., Koczywas M., Forster M., Cameron R.B. (2022). EZH2 Inhibitor Tazemetostat in Patients with Relapsed or Refractory, BAP1-Inactivated Malignant Pleural Mesothelioma: A Multicentre, Open-Label, Phase 2 Study. Lancet Oncol..

[176] Tang M., Gong M., Liu X., Zhang T., Liu Z., Song D. (2025). Recent Update on the Development of EZH2 Inhibitors and Degraders for Cancer Therapy. Eur. J. Med. Chem..

[177] Zingg D., Arenas-Ramirez N., Sahin D., Rosalia R.A., Antunes A.T., Haeusel J., Sommer L., Boyman O. (2017). The Histone Methyltransferase Ezh2 Controls Mechanisms of Adaptive Resistance to Tumor Immunotherapy. Cell Rep..

[178] Zhou L., Mudianto T., Ma X., Riley R., Uppaluri R. (2020). Targeting EZH2 Enhances Antigen Presentation, Antitumor Immunity, and Circumvents Anti-PD-1 Resistance in Head and Neck Cancer. Clin. Cancer Res..

[179] Morel K.L., Sheahan A.V., Burkhart D.L., Baca S.C., Boufaied N., Liu Y., Qiu X., Cañadas I., Roehle K., Heckler M. (2021). EZH2 Inhibition Activates a dsRNA-STING-Interferon Stress Axis That Potentiates Response to PD-1 Checkpoint Blockade in Prostate Cancer. Nat. Cancer.

[180] Mortezaee K. (2025). EZH2 Regulatory Roles in Cancer Immunity and Immunotherapy. Pathol. Res. Pract..

[181] Piunti A., Meghani K., Yu Y., Robertson A.G., Podojil J.R., McLaughlin K.A., You Z., Fantini D., Chiang M., Luo Y. (2022). Immune Activation Is Essential for the Antitumor Activity of EZH2 Inhibition in Urothelial Carcinoma. Sci. Adv..

[182] Chibaya L., Murphy K.C., DeMarco K.D., Gopalan S., Liu H., Parikh C.N., Lopez-Diaz Y., Faulkner M., Li J., Morris J.P. (2023). EZH2 Inhibition Remodels the Inflammatory Senescence-Associated Secretory Phenotype to Potentiate Pancreatic Cancer Immune Surveillance. Nat. Cancer.

[183] Nagarsheth N., Peng D., Kryczek I., Wu K., Li W., Zhao E., Zhao L., Wei S., Frankel T., Vatan L. (2016). PRC2 Epigenetically Silences Th1-Type Chemokines to Suppress Effector T-Cell Trafficking in Colon Cancer. Cancer Res..

[184] Bugide S., Green M.R., Wajapeyee N. (2018). Inhibition of Enhancer of Zeste Homolog 2 (EZH2) Induces Natural Killer Cell-Mediated Eradication of Hepatocellular Carcinoma Cells. Proc. Natl. Acad. Sci. USA.

[185] Zhong J., Yang X., Chen J., He K., Gao X., Wu X., Zhang M., Zhou H., Xiao F., An L. (2022). Circular EZH2-Encoded EZH2-92aa Mediates Immune Evasion in Glioblastoma via Inhibition of Surface NKG2D Ligands. Nat. Commun..

[186] Huang S., Wang Z., Zhou J., Huang J., Zhou L., Luo J., Wan Y.Y., Long H., Zhu B. (2019). EZH2 Inhibitor GSK126 Suppresses Antitumor Immunity by Driving Production of Myeloid-Derived Suppressor Cells. Cancer Res..

[187] Mola S., Pinton G., Erreni M., Corazzari M., De Andrea M., Grolla A.A., Martini V., Moro L., Porta C. (2021). Inhibition of the Histone Methyltransferase EZH2 Enhances Protumor Monocyte Recruitment in Human Mesothelioma Spheroids. Int. J. Mol. Sci..

[188] Wang Y., Yu L., Hu Z., Fang Y., Shen Y., Song M., Chen Y. (2022). Regulation of CCL2 by EZH2 Affects Tumor-Associated Macrophages Polarization and Infiltration in Breast Cancer. Cell Death Dis..

[189] Liu J., Lin W.-P., Su W., Wu Z.-Z., Yang Q.-C., Wang S., Sun T.-G., Huang C.-F., Wang X.-L., Sun Z.-J. (2023). Sunitinib Attenuates Reactive MDSCs Enhancing Anti-Tumor Immunity in HNSCC. Int. Immunopharmacol..

[190] Dumoulin D.W., Cornelissen R., Bezemer K., Baart S.J., Aerts J.G.J.V. (2021). Long-Term Follow-Up of Mesothelioma Patients Treated with Dendritic Cell Therapy in Three Phase I/II Trials. Vaccines.

[191] Aerts J.G., Belderbos R., Baas P., Scherpereel A., Bezemer K., Enninga I., Meijer R., Willemsen M., Berardi R., Fennell D. (2024). Dendritic Cells Loaded with Allogeneic Tumour Cell Lysate plus Best Supportive Care versus Best Supportive Care Alone in Patients with Pleural Mesothelioma as Maintenance Therapy after Chemotherapy (DENIM): A Multicentre, Open-Label, Randomised, Phase 2/3 Study. Lancet Oncol..

[192] Klampatsa A., Albelda S.M. (2020). Current Advances in CAR T Cell Therapy for Malignant Mesothelioma. J. Cell. Immunol..

[193] Morello A., Sadelain M., Adusumilli P.S. (2016). Mesothelin-Targeted CARs: Driving T Cells to Solid Tumors. Cancer Discov..

[194] Wang Y., Zhao G., Xing S., Wang S., Li N. (2024). Breaking through the Treatment Desert of Conventional Mesothelin-Targeted CAR-T Cell Therapy for Malignant Mesothelioma: A Glimpse into the Future. Pharmacol. Res..

[195] Chalise L., Kato A., Ohno M., Maeda S., Yamamichi A., Kuramitsu S., Shiina S., Takahashi H., Ozone S., Yamaguchi J. (2022). Efficacy of Cancer-Specific Anti-Podoplanin CAR-T Cells and Oncolytic Herpes Virus G47Δ Combination Therapy against Glioblastoma. Mol. Ther. Oncolytics.

[196] Pease D.F., Kratzke R.A. (2017). Oncolytic Viral Therapy for Mesothelioma. Front. Oncol..

[197] Kuryk L., Haavisto E., Garofalo M., Capasso C., Hirvinen M., Pesonen S., Ranki T., Vassilev L., Cerullo V. (2016). Synergistic Anti-Tumor Efficacy of Immunogenic Adenovirus ONCOS-102 (Ad5/3-D24-GM-CSF) and Standard of Care Chemotherapy in Preclinical Mesothelioma Model. Int. J. Cancer.

[198] Di Somma S., Iannuzzi C.A., Passaro C., Forte I.M., Iannone R., Gigantino V., Indovina P., Botti G., Giordano A., Formisano P. (2019). The Oncolytic Virus Dl922-947 Triggers Immunogenic Cell Death in Mesothelioma and Reduces Xenograft Growth. Front. Oncol..

[199] Ponce S., Cedrés S., Ricordel C., Isambert N., Viteri S., Herrera-Juarez M., Martinez-Marti A., Navarro A., Lederlin M., Serres X. (2023). ONCOS-102 plus Pemetrexed and Platinum Chemotherapy in Malignant Pleural Mesothelioma: A Randomized Phase 2 Study Investigating Clinical Outcomes and the Tumor Microenvironment. J. Immunother. Cancer.

[200] Chesney J.A., Ribas A., Long G.V., Kirkwood J.M., Dummer R., Puzanov I., Hoeller C., Gajewski T.F., Gutzmer R., Rutkowski P. (2023). Randomized, Double-Blind, Placebo-Controlled, Global Phase III Trial of Talimogene Laherparepvec Combined with Pembrolizumab for Advanced Melanoma. J. Clin. Oncol..

[201] Patel R.B., Hernandez R., Carlson P., Grudzinski J., Bates A.M., Jagodinsky J.C., Erbe A., Marsh I.R., Arthur I., Aluicio-Sarduy E. (2021). Low-Dose Targeted Radionuclide Therapy Renders Immunologically Cold Tumors Responsive to Immune Checkpoint Blockade. Sci. Transl. Med..

[202] Robert C., Gastman B., Gogas H., Rutkowski P., Long G.V., Chaney M.F., Joshi H., Lin Y.-L., Snyder W., Chesney J.A. (2024). Open-Label, Phase II Study of Talimogene Laherparepvec plus Pembrolizumab for the Treatment of Advanced Melanoma That Progressed on Prior Anti-PD-1 Therapy: MASTERKEY-115. Eur. J. Cancer.

[203] Revelant A., Gessoni F., Montico M., Dhibi R., Brisotto G., Casarotto M., Zanchetta M., Paduano V., Sperti F., Evangelista C. (2025). Radical Hemithorax Radiotherapy Induces an Increase in Circulating PD-1+ T Lymphocytes and in the Soluble Levels of PD-L1 in Malignant Pleural Mesothelioma Patients: A Possible Synergy with PD-1/PD-L1 Targeting Treatment?. Front. Immunol..

[204] Cascone T., Awad M.M., Spicer J.D., He J., Lu S., Sepesi B., Tanaka F., Taube J.M., Cornelissen R., Havel L. (2024). Perioperative Nivolumab in Resectable Lung Cancer. N. Engl. J. Med..

[205] Wakelee H., Liberman M., Kato T., Tsuboi M., Lee S.-H., Gao S., Chen K.-N., Dooms C., Majem M., Eigendorff E. (2023). Perioperative Pembrolizumab for Early-Stage Non–Small-Cell Lung Cancer. N. Engl. J. Med..

[206] Forde P.M., Spicer J., Lu S., Provencio M., Mitsudomi T., Awad M.M., Felip E., Broderick S.R., Brahmer J.R., Swanson S.J. (2022). Neoadjuvant Nivolumab plus Chemotherapy in Resectable Lung Cancer. N. Engl. J. Med..

[207] Heymach J.V., Harpole D., Mitsudomi T., Taube J.M., Galffy G., Hochmair M., Winder T., Zukov R., Garbaos G., Gao S. (2023). Perioperative Durvalumab for Resectable Non–Small-Cell Lung Cancer. N. Engl. J. Med..

[208] Ambrosini P., Stanzi A., Lo Russo G., Solli P., Occhipinti M. (2025). Towards a New Approach in Pleural Mesothelioma: Perioperative Immunotherapy and Its Implications. Crit. Rev. Oncol. Hematol..

[209] Cecchi L.G., Aliprandi M., De Vincenzo F., Perrino M., Cordua N., Borea F., Bertocchi A., Federico A., Marulli G., Santoro A. (2025). Perioperative Treatments in Pleural Mesothelioma: State of the Art and Future Directions. Cancers.

[210] Kong S.L., Feng Z., Kim S., Ha E.K., Kamel K., Becich M., Luketich J.D., Pennathur A. (2025). Hyperthermic Intrathoracic Chemoperfusion and the Role of Adjunct Immunotherapy for the Treatment of Pleural Mesothelioma. Biomolecules.

